# Active waveguide Bragg lasers via conformal contact PDMS stamps

**DOI:** 10.1038/s41598-022-26218-7

**Published:** 2022-12-23

**Authors:** Yun Li, Girish Lakhwani

**Affiliations:** 1 ARC Centre of Excellence in Exciton Science, School of Chemistry, The University of Sydney, Sydney, NSW 2006 Australia; 2 The University of Sydney Nano Institute, Sydney, NSW 2006 Australia; 3 Institute of Photonics and Optical Science, The University of Sydney, Sydney, NSW 2006 Australia

**Keywords:** Lasers, LEDs and light sources, Optical materials and structures, Surface patterning

## Abstract

Lasing is observed in Bragg lasers formed through conformal contact of a patterned PDMS stamp with a plain active film, spincoated on glass. The thresholds, output efficiencies and spectral characteristics are compared to standard substrate patterned gratings and is discussed in relation to the coupling coefficient $\upkappa$. The reported thresholds are highly sensitive in distributed feedback (DFB) lasers to grating duty cycles, for both PDMS-air and substrate–film lasers. Overall, laser thresholds of PDMS-air (PA) DFB lasers are found to be significantly higher than substrate–film (SF) lasers, which is attributed to an approximate three-fold reduction of optical-confinement in the grating region. Slope output efficiencies are found to be comparatively higher in PA lasers relative to SF lasers for both DFB and DBR configurations and is attributed to several competing factors. The PDMS can be removed from the surface of the active film repeatedly and conformal contact is limited mainly by the particle build up on the PDMS surface. The proposed PA system is expected to be useful in rapid laser metrology of new gain materials and in practical applications of optically pumped lasers.

## Introduction

Solution processed lasers^1–3^ have seen significant progress in recent years and offer solutions to low-cost, simple fabrication, tunable light sources for myriad of applications including integrated lab-on-chip devices, spectroscopy, and sensing. For practical applications, compact electrically driven lasers are desired. However major hurdles currently stunt progress into electrically injected lasers^4^. In the case of organic semiconductor electrically injected lasers, statistical triplet exciton formation limits the inversion density and introduces excited-state absorption loss, while accompanied by losses from injection electrodes. In addition, degradation at the high excitation densities required to achieve lasing must be addressed if such devices are to ever see commercialization. The above-mentioned issues can be circumvented if instead, the organic semiconductor laser is optically pumped above the threshold by an electrically driven InGaN laser diode^5–9^. In this configuration, while the overall cost and compactness is limited by the requirement of a secondary laser, it retains the advantages of the organic semiconductor material, and compactness.

Reports on optically pumped solution-processed organic DFB lasers have comprised primarily of substrate-defined corrugations, active layer corrugations via nanoimprint and active films defined on patterned flexible stamps^8–10^. The latter two cases are desirable to further reduce the fabrication cost. However, in most of these cases, significant periodic modulation in active-layer thickness is present, resulting in a corresponding modulation in optical confinement. This can result in complex, mixed, gain/refractive index coupled distributed feedback^11,12^. Moreover, the waveguide mode is highly sensitive to defects in the corrugated structure of spin-coated films.

Recent reports suggest that these issues can be circumvented by patterning the resonators above the active layer^13,14^. In this geometry, modulation in active layer thickness is absent, and the waveguide mode profile has been shown to be relatively unperturbed by defects in the resonator^14^. However, patterning corrugations in this way is challenging as the active film is susceptible to damage from the fabrication process. Common fabrication techniques for achieving active layer gratings involve holographic patterning onto photoresist, which can result in potential active layer damage. Nevertheless, lasers fabricated in this way have shown lower lasing thresholds and higher output slope efficiencies^13,15^.

**Figure 1:**
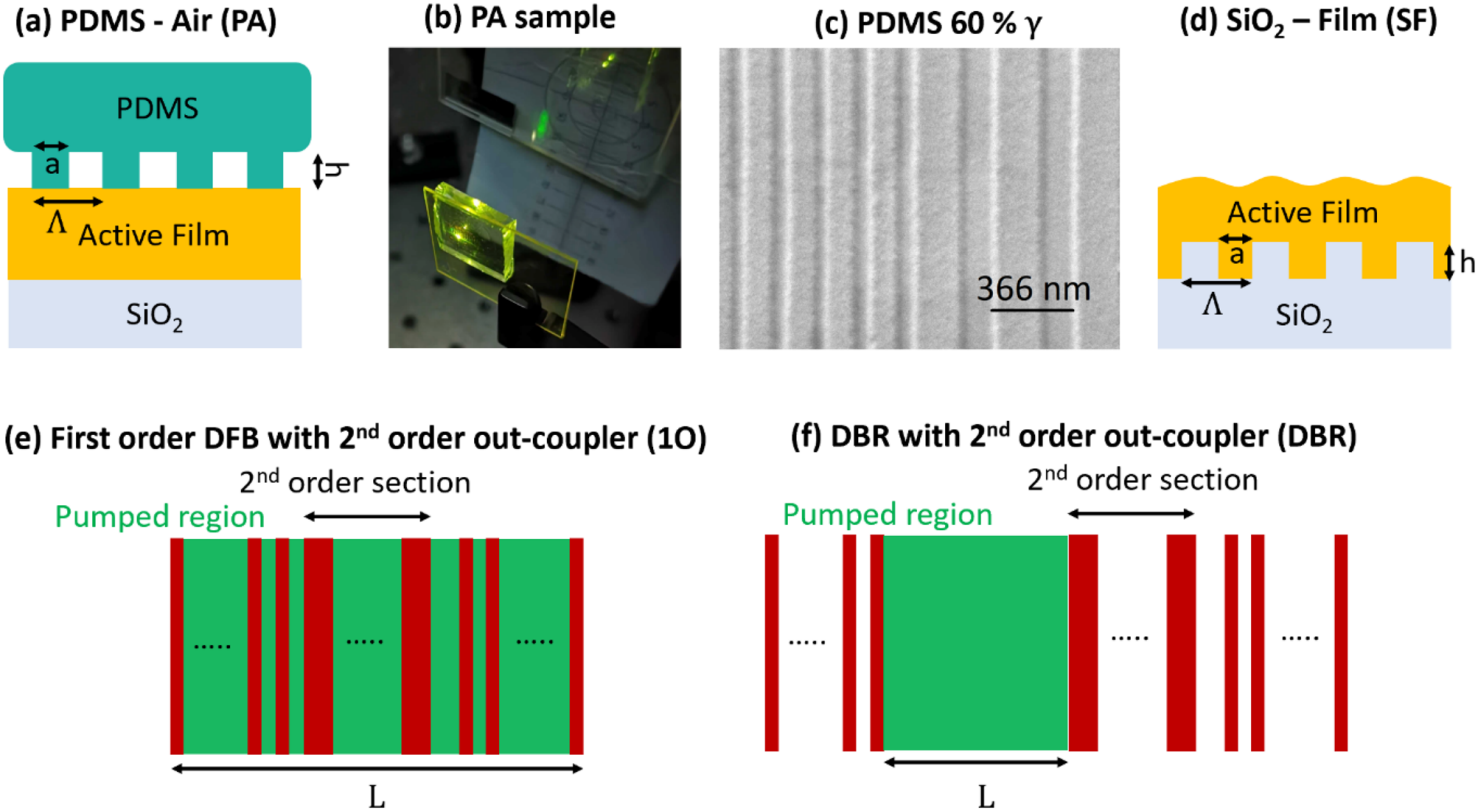
(**a**) Schematic for PDMS—air (PA) grating, (**b**) Pictogram of lasing from PA sample (pump beam filtered), (**c**) SEM of 60% γ 1st and 2nd order PDMS gratings, (**d**) schematic for substrate—film (SF) grating, (**e**) 1O DFB laser with 40 periods of a 2nd order grating out-coupler between 1st order gratings (**f**) 1O DBR laser with 1st order gratings and 40 periods of 2nd order grating out-coupler defined on a single mirror facet.

In this report, lasing is demonstrated by bringing a patterned PDMS stamp in conformal contact with an active film (Fig. 1a–c) to form active-layer corrugations. In this configuration, feedback reflections are provided by the PDMS-air (PA) grating and the resonator can be detached from the active film. Although flexible stamps are commonly used to directly imprint corrugations for lasing, we forego this step and use the stamp to directly achieve lasing. Due to the low surface energy of PDMS, damage to the active film is minimal, and the PDMS can be peeled on/off repeatedly with no deleterious effect on lasing performance. The longevity of the sample is determined primarily by dust/particles accumulation on the PDMS surface. For material systems suffering from photo-degradation, the laser can be recovered by moving the stamp to a different location on the active film. Additionally, the system can be useful for the qualification of gain/lasing performance in materials and films without expensive/repetitive fabrication steps beyond the initial stamp fabrication. Here, the performance of the proposed PA Bragg lasers is compared to standard substrate–film (SF) (Fig. 1d) Bragg lasers, with respect to lasing thresholds and output efficiencies.

## Results/Discussion

In this study, F8_0.9_BT_0.1_ (ADS233YE) was used for its commercial availability and broadband gain spectrum^16^. The latter is important to minimize variability in lasing thresholds due to changes in effective refractive index (${n}_{eff}$) away from the peak of the gain spectrum between PA and SF structures. The native active film thickness was fixed at 180 nm for all samples; we find this is a sufficient compromise to obtain an appreciable pump-mode overlap and moderate optical confinement^17^. The film thickness was low enough that only the TE_0_ mode propagates with substantial confinement. Pure 2nd order (2O) lasers are commonly used due to less stringent fabrication requirements than 1st order lasers, and ease of metrology since the laser emission is vertically outcoupled. However 1st order lasers tend to yield lower thresholds, as the maximum theoretical diffraction efficiency for feedback is stronger than 2nd order lasers for optimized duty cycles $\gamma =\frac{a}{\Lambda }$ , where $a$ is the grating linewidth and $\Lambda$ is the periodicity, as illustrated in Fig. 1a,d^18–20^. To retain both high feedback strength and vertical out-coupling, lasers made from 1st order gratings with a 2nd order out-coupler (1O) have been used^21^. Here, 2O DFB, 1O DFB and DBR lasers are explored for both PA and SF structures.

**Figure 2:**
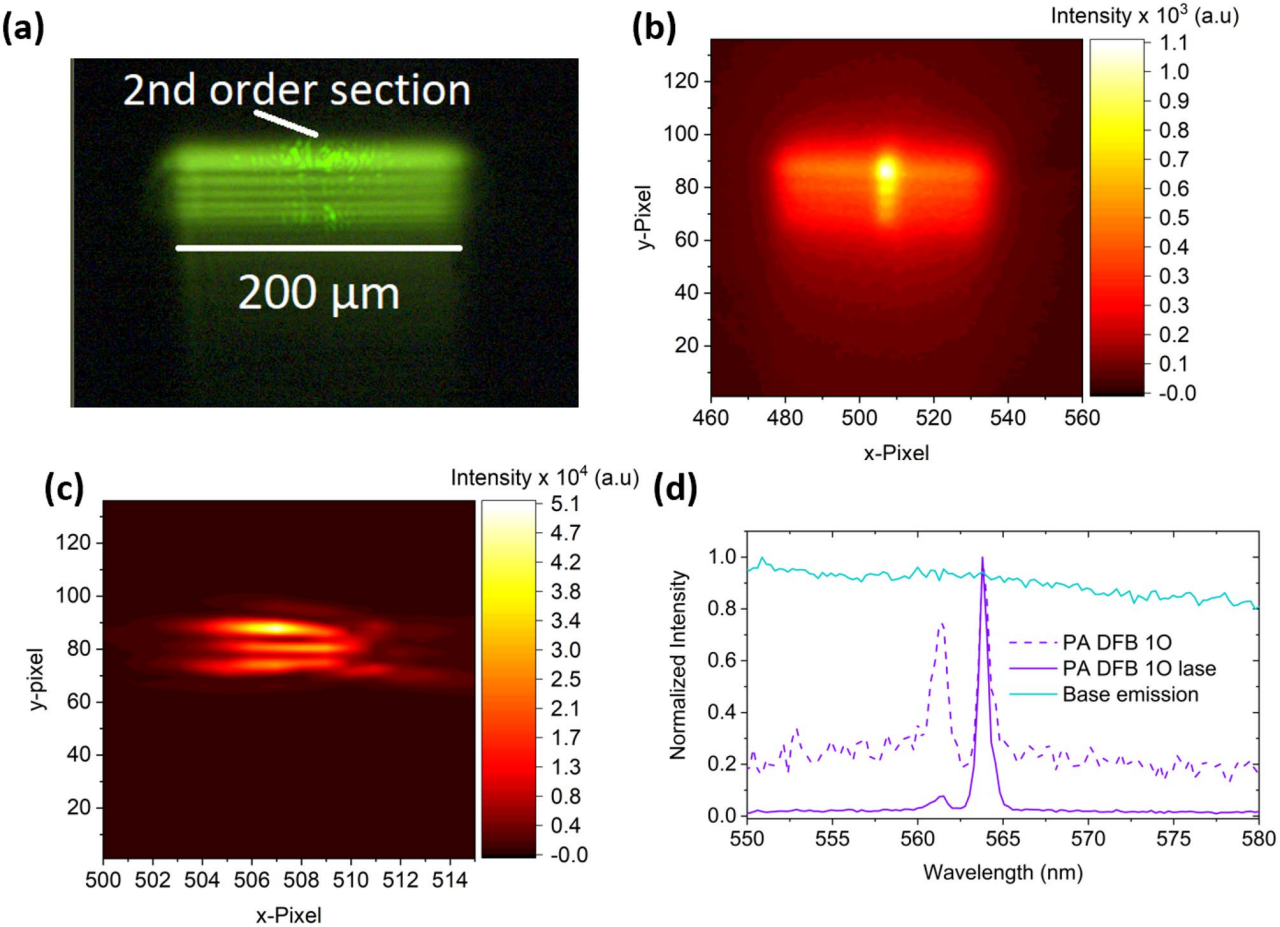
(**a**) zoom lens image of PA 1O DFB operated above threshold. Magnified (~ 4 ×) spatial “nearfield” captured by fully open entrance slit spectrograph in 0th order diffraction (reflection mode) of 60% $\gamma$ PA 1O DFB (**b**) below and (**c**) above threshold. (**d**) Normalized spectra for 60% $\gamma$ PA 1O DFB laser below lasing threshold (dotted violet) and above lasing threshold (filled violet), along with base emission in the absence of a grating.

For 1O DFB lasers, 40 periods of 2nd order gratings were placed between two 1st order gratings (Fig. 1e). For 1O DBR lasers, 500 1st order periods are used for both mirrors; this was sufficient to achieve full reflection of the waveguided light, while 40 2nd order periods were placed on one mirror facet for outcoupling (Fig. 1f). Gratings of periodicity of $\Lambda$ = 366, 183 nm were chosen for 2nd/1st order gratings to match the Bragg condition, $m{\lambda }_{0}={2n}_{eff}\Lambda$, for wavelength, ${\lambda }_{0}$~ 565 nm (near peak of gain bandwidth based on amplified spontaneous emission (ASE)^16^) corresponding to ${n}_{eff}$ ~ 1.53. All cavity lengths were fixed to approximately 200 µm, including 2O DFB lasers, and excitation stripe is shaped to ~ 200 µm × 50 µm, as described in Fig. S7, to match the cavity dimension and shown with a zoom lens image (Fig. 2a).

### Observation of lasing in PDMS-air laser

Scattered laser radiation in Fig. 2a could be observed from the grating above threshold and the outline of the 2nd order out-coupler is visible, however, the vertically outcoupled emission was not observed as the image is taken at oblique incidence. Magnified (~ 3.8 × , by comparing physical stripe length and spectrograph image) 0th order diffraction (reflection mode, fully open entrance slit) spectrograph images of the emission for a 1O DFB below and above threshold are illustrated in Fig. 2b,c respectively. The vertically out-coupled emission from the 2nd order section is discernible from the background emission of the excitation stripe below threshold. Above threshold, emission is predominantly localized in the 2nd order section, and spans ~ 5 pixels (65 µm), and similarly for 1O DBR samples (Fig. S9a–d). All spectra were taken with a 50 µm entrance slit, therefore we expect that most of the lasing emission (~ 77%) was captured with 1O DFB and DBR samples.

Normalized spectra for base film emission are given in Fig. 2d) along with spectra for 60% $\gamma$ PA 1O DFB below and above threshold. Below threshold, two sharp peaks close to the minimum spectral resolution of the spectrograph (~ 0.7 nm) was observed, along with a dip in the spectral intensity between the two peaks at 563 nm. The spectral position of this dip is relatively close to the fundamental TE_0_ mode (numerically calculated ${n}_{eff} \sim 1.52$, depending on grating $\gamma$ as shown in Fig. S10), thus we assign it to the photonic stopband of the TE_0_ mode. Also shown is the lasing peak for the low energy longitudinal mode, though we find that the oscillating mode can begin at the high energy peak and will lase at both modes with higher pump fluence (Fig. S11a). This is expected, as there is no mode threshold discrimination process in 1st order DFB lasers unlike 2O DFB lasers^22–24^. In 1st order lasers, the threshold gain for each mode nearest the stopband are equally likely to lase in the absence of a defect/phase-shifting element^24^. For 2O DFB lasers, mode discrimination is provided by differential radiation loss of either mode^25^. Nevertheless, we do see some level of discrimination between repeated samples for the 1O DFB laser. This is attributed to a multitude of factors including, differential loss/gain at different wavelengths, altered grating phase due to the 2nd order grating out-coupler, or small reflections from adjacent gratings^26^.

### Fluence dependant lasing output and slope output efficiency in PA laser

**Figure 3:**
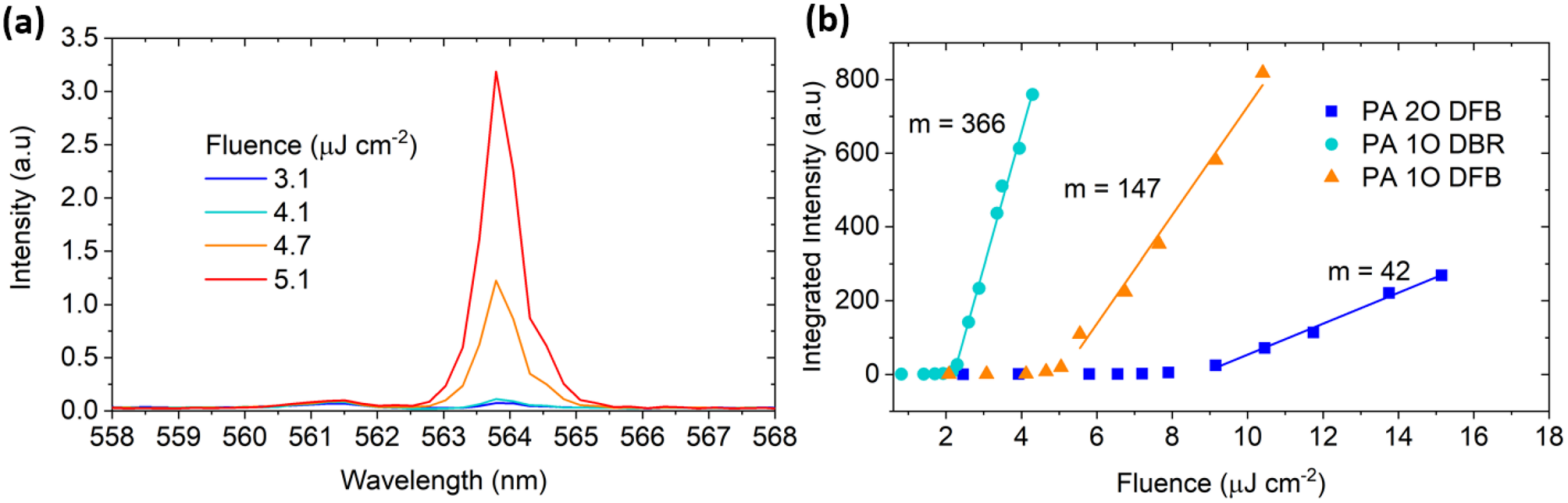
(**a**) Fluence dependant spectra for 60% $\gamma$ PA 1O DFB, (**b**) typical fluence dependant output of integrated spectral output near the lasing wavelength for 60% $\gamma$ PA 1O DBR/ DFB and 2O DFB lasers, with slope efficiency, m shown.

Fluence-dependant spectra of 60% $\gamma$ PA 1O DFB sample is illustrated in Fig. 3a, a super-linear growth of the low energy band-edge feature was observed with increasing fluence indicating the onset of lasing. Similar trends in intensity growth were observed in 2O DFB and 1O DBR lasers (Fig. S12). Growth of the integrated spectral intensity near the lasing peak (± 10 nm) with fluence is shown in Fig. 3b for a typical set of 60% $\gamma$ PA 1O DFB, 1O DBR and 2O DFB samples.

Lowest thresholds were observed in 1O DBR samples, corresponding, also to the highest output efficiency, followed by the 1O DFB sample. The highest threshold belongs to the 2O DFB laser, with lowest apparent slope efficiency. However, the low slope efficiency of the 2O DFB laser can be mainly attributed to its large spatial emission area (Fig. S9f.), resulting in a large proportion of light that was not collected by the 50 µm spectrograph entrance slit. For 2O DFB lasers, the higher relative thresholds can be partially attributed to reduced feedback as later discussed and an increase in outcoupling loss. For 1O DFB and DBR lasers, since the two operate by different physical mechanisms, direct comparisons of thresholds are difficult. For DBR lasers, the active gain medium is separate from the periodic element. The Bragg reflectors act as mirrors and the DBR lasers behave as spectrally selective Fabry–Perot lasers and lasing occurs within the stopband, where the reflectivity is the highest. In DFB lasers, the gain medium is integrated with the periodic element, and feedback occurs via periodic reflection of counter-propagating waves at the band-edges.

### Spectral and lasing properties as a function of duty cycle for PA and SF lasers

To further explore the discrepancy in thresholds, we look at the general expression derived from coupled-mode theory for the coupling coefficient of a pure index-coupled laser, assuming a perfectly square- periodic profile^11,19,20^,

1 $$ \begin{array}{*{20}c} {\kappa \propto \left| {\frac{{k_{0} \left( {n_{2}^{2} - n_{1}^{2} } \right)\Gamma_{g} }}{{2n_{eff} \pi m}}\sin \left( { \pi m\gamma } \right)} \right| \propto \frac{\Delta \nu }{2}.} \\ \end{array} $$

Here, ${\mathrm{k}}_{0}=\frac{2\pi }{{\lambda }_{0}}$ where ${\lambda }_{0}$ is the propagation wavelength in free space, ${n}_{2} ,{n}_{1}$ are the refractive indices of the grating materials (SF/PA), ${\Gamma }_{g}$ is the modal confinement in the grating region, ${n}_{eff}$ is the effective refractive index, *m* is the Bragg order, a is the grating linewidth and $\mathrm{\Delta \nu }$ is the longitudinal mode spacing at the photonic band edges. We make a point here that the equation is derived with a perturbative approach assuming that the refractive index contrast between ${n}_{2}$ and ${n}_{1}$ is small compared to ${n}_{eff}$. Therefore, quantitatively, it is not directly applicable to DFB lasers comprised of solution processed materials where the index contrast is typically high, and the active layer refractive index is low. Nevertheless, we instead use Eq. (1) to qualitatively predict the behaviour of the PA samples with comparison to standard SF samples.

Note that $\gamma$ used in this context refers to the initial design pattern dimensions of positive e-beam resist for lithography, and not the exact physical ratio of the linewidth to the grating periodicity. This is because the linewidths will depend on e-beam exposure dosage and other practical fabrication factors. The PDMS used in the PA sample was moulded from the same SiO_2_ gratings used in SF samples. For SF samples, the grating corrugations were smoothened out such that the topology of the film surface was only modulated by at most 10 nm (Fig. S13, S14). This results in an optical confinement modulation of ~ 0.23 (Fig. S10a), assuming the active film thickness is 130 nm in the grating troughs and 180 nm in the trenches. We can therefore expect a large gain-coupling contribution from the periodic modulation in confinement for DFBs with the SF samples in addition to the index coupling. In comparison, the optical confinement in the active film for PA samples is virtually unchanged (Fig. S10b) since there is no modulation in active film thickness.

**Figure 4:**
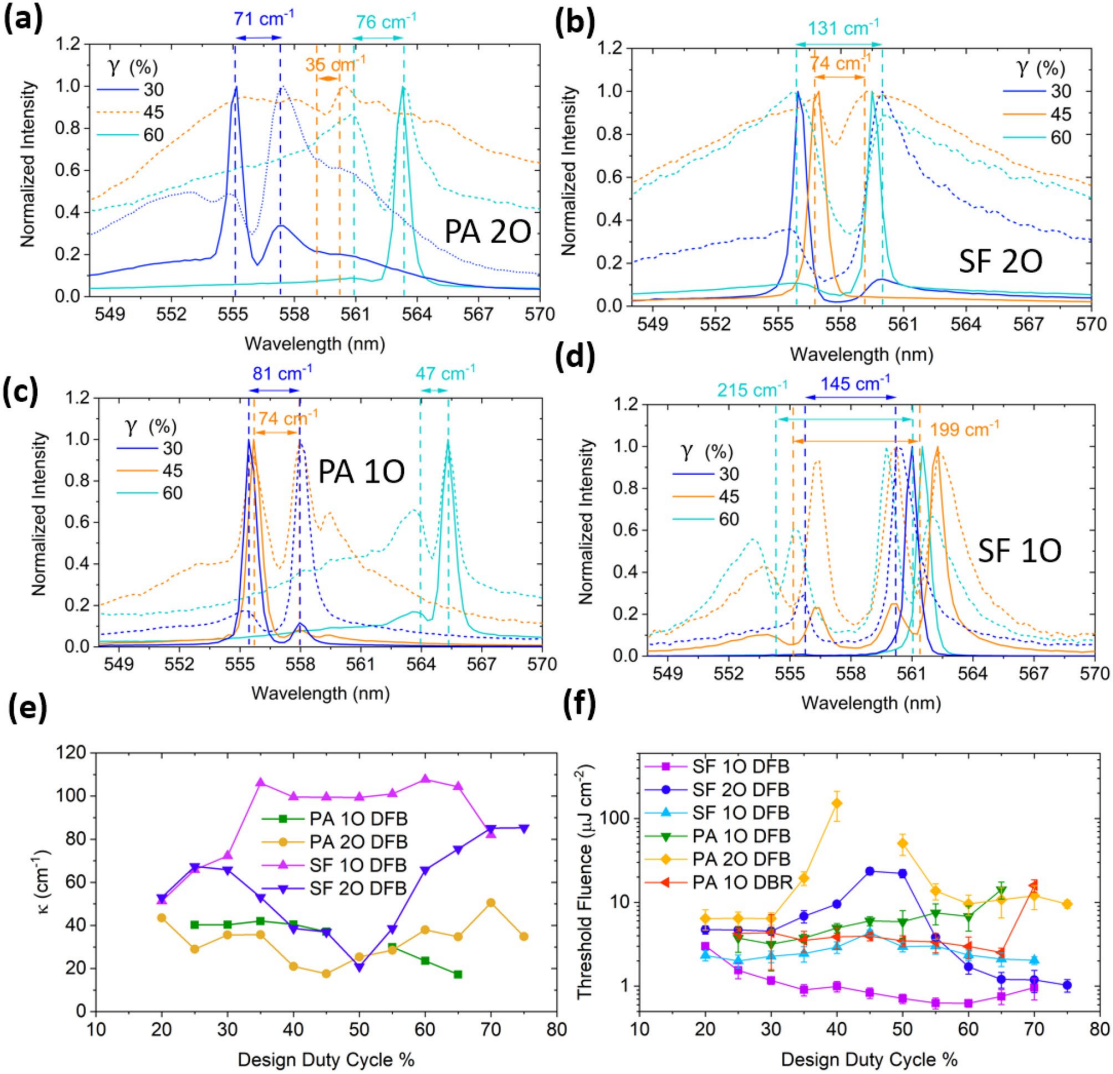
Spectra for above lasing threshold (solid line) and below lasing threshold (dashed line) with stopband widths for 30, 45, 60% $\gamma$ of (**a**) PA 2O, (**b**) SF 2O, (**c**) PA 1O, (**d**) SF 1O DFB lasers. (**e**) Calculated coupling coefficients based on measured stopband widths of DFB lasers. (**f**) Threshold fluence dependence on design duty cycle for 1O DBR, 1O DFB and 2O samples for both substrate–film (SF) and PDMS-air (PA) gratings lasers.

Figure 4a–d show representative experimental spectra for SF, PA 1O and 2O DFB samples of 30, 45 and 60% $\gamma$ above and below lasing threshold. The stopband widths $\mathrm{\Delta \nu }$ are annotated in energy units, and were used to estimate the coupling coefficients according to Eq. (1). For PA 2O stopbands are clearly observed for 30, 60% $\gamma$, however, for 45% $\gamma$, the dip was less prominent, with the stopband width noticeably narrower and similarly for SF 2O in Fig. 4b. Additionally, lasing was not observed in the 45% $\gamma$ PA sample at the highest fluences before the film was ablated, otherwise lasing was observed on either side of the stopband. The observations are consistent with the $\mathrm{sin}\left(\pi m\gamma \right)$ term in Eq. (1) for $m=2.$ Close to $\gamma =0.5$, the coupling coefficient is null $\upkappa =0$, therefore little or no coupling is expected close to this $\gamma$, while $\upkappa$ is at its highest at 25, 75% $\gamma$. Practically, deviation from a perfectly square profile, will result in an incomplete null of $\upkappa$^26^. Conversely, for 50% $\gamma$ in 1O samples, $m=1$, $\upkappa$ reaches its maximum value, and departure from 50% $\gamma$ results in a relatively slowly decreasing $\upkappa$.

Additional peaks and dips on either side of the main stopband for 1O samples were observed, with the dips more prominent for SF samples shown in Fig. 4c,d, particularly for 45% $\gamma$ PA 1O DFB , and 45, 60% $\gamma$ SF 1O DFB. We preclude the possibility of the higher order TE modes and TM modes based on mode-solver calculations of ${n}_{eff}$ and the predicted spectral position from the Bragg equation (TM_0_ spectral feature is assigned in Fig. S15).

The symmetric distribution of the peaks away from the main stopband suggests these may be the sidemodes found in typical Bragg structures^26^. Inspection of Fig. 4d suggests that the dips form directly from the band-edge peaks. At 30% $\gamma$, no obvious dips are present, and the band-edge mode intensity is skewed to the high-wavelength edge. However, for 45% and 60% $\gamma$, new dips appear to emerge from both band-edge peaks (the transition is more clearly observed in Fig. S16a,e), and the intensity of the high-wavelength band-edge peak decreases relative to the low wavelength edge. Moreover, the assignment of these dips to sidemodes, would suggest that the main stopband width decreases as $\gamma$ hovers around 50% $\gamma$, where $\upkappa$ is expected to reach its maxima.

For 45% $\gamma$ PA 1O DFBs, lasing still occurs within the main stopband, however, for SF 1O samples, lasing appears to occur in the high wavelength side-dip/band. Assuming that the new dips split directly from the main band-edge modes, the centre of the side-dips were used to calculate $\upkappa$. Where the position of the dips is ambiguous, as with 45% $\gamma$ PA 1O, the main stopband was used to calculate the width, noting some underestimation of $\upkappa$ values. Even if calculated with the side bands, we find consistently lower $\mathrm{\Delta \nu }$ for PA compared to SF for both 1O and 2O DFB samples and thus correspondingly lower $\upkappa$ for all $\gamma$ shown in Fig. 4e. Minima/maximum in both PA and SF 2O DFBs were observed at around 50/25 and 75% $\gamma$, agreeing relatively well with Eq. (1). A less discernible trend was observed for the 1O samples close to 50% $\gamma$; however, we attribute this mainly to the ambiguity in spectral positions of the Bragg dips and fabrication limitations of PDMS replication for $\gamma$ at the extremities. Overall, the appearance of the side-dips appears to correlate with high coupling coefficients in 1O but not 2O lasers; however, the origin of the features are currently unknown.

We observe that trends in DFB lasing thresholds follow trends in $\upkappa$ closely, as shown in Fig. 4f, that is, lower thresholds for higher $\upkappa$. Thresholds were obtained by averaging over at least 3 test samples. The lowest thresholds obtained were 0.63 µJ cm^−2^ for 55% $\gamma$ 1O SF DFB samples and 1.01 µJ cm^−2^ for 75% $\gamma$ 2O SF DFB. The threshold could be further reduced to 0.45 µJ cm^−2^ in 1O SF DFBs by replacing the 2nd order grating of the same $\gamma$ with a 75% $\gamma$ 2nd order grating (Fig. S17b). On the other hand, the highest thresholds obtained are 23.5 µJ cm^−2^ for 45% $\gamma$ 2O SF, and > 300 µJ cm^−2^ for 45% $\gamma$ 2O PA (threshold not reached before film ablation). The results show that a poorly optimized $\gamma$ could raise the lasing thresholds by over an order of magnitude for 2O lasers. Improved performance of 1st order lasers with 2nd order out-couplers in previous reports^21^, can therefore be attributed to, at least in part, to unoptimized grating duty cycles.

Contrary to previous reports of lower lasing thresholds in surface-corrugated lasers relative to SF lasers by Quintana et al.^15^, significantly higher thresholds were observed here inin the former. We suspect that this is partially due to the different excitation and resonator lengths used. In our work, the excitation stripe and resonator lengths are exactly matched to 200 µm. We’ve shown that the thresholds can be further reduced by increasing the total feedback with longer resonators and correspondingly, longer stripe lengths (Fig. S18), in line with theoretical predictions^11^. The reduction in threshold tapers off with higher cavity length, however, the saturation occurs later for PA lasers than SF lasers due to the lower κ. For example, an approximate 2.7-fold reduction in threshold for PA 2O lasers was found when increasing the cavity length from 200 to 400 µm, whereas only a 1.2-fold reduction was observed in SF 2O lasers.

In work by Quintana et al., the holographically patterned gratings presumably encompass a larger area than the excitation stripe length used (1100 µm). We expect with these large resonator/excitation lengths, the thresholds are relatively saturated. Nevertheless, we find that even at longer cavity lengths, thresholds in PA lasers are consistently higher than in SF lasers. Instead, we mainly attribute the higher lasing thresholds of PA lasers for all γ, primarily to an approximate threefold reduction in ${\Gamma }_{g}$ (${\Gamma }_{g}$ ~ 0.2 for SF compared to ~ 0.07 for PA, depending on $\gamma$, calculated as shown in Fig. S19 and given in Table S1), and correspondingly, a reduction in $\upkappa$. In contrast, the SF lasers by Quintana et al. have used dye-doped polystyrene (refractive index ~ 1.59 at the lasing wavelength) matrices as the active layer with DCG/SiO_2_ (refractive index 1.55/1.46) gratings, resulting in significantly lower index modulation (1.59–1.55/1.59–1.46) compared to DCG-air active-layer surface corrugation lasers. The reduction of ${\Gamma }_{g}$ in PA lasers appears to outweigh any increase in $\upkappa$ due to higher grating index contrast (1.43–1 compared to 1.7–1.46), and lower thresholds due to higher confinement in the active layer relative to SF lasers. However, for the SF lasers, differences in thresholds may also be attributed to contributions from gain-coupling from the modulation in confinement. ${\Gamma }_{g}$ can be increased by reducing the active layer thickness and/or refractive index, thereby increasing the overlap of the evanescent portion of the mode. However, this would also result in a decrease in confinement in the active layer. In previous work, the confinement around the upper active film region could be increased by depositing a low loss, high dielectric constant material atop the active film^27^. In this case, the overall confinement in the active film would increase only if the film thickness was kept thin.

For DBR lasers, the thresholds for PA and SF lasers were comparable, implying that the thresholds are not strongly correlated with $\upkappa .$ We attribute this to a combination of low waveguide loss (~ 11 cm^−1^ as determined in Fig. S20) and complete reflection from the mirrors. Although lower $\upkappa$ may increase the penetration depth into the DBR mirrors, assuming the loss upon round-trip reflection remains relatively unchanged, it would not significantly alter laser feedback for the same gain.

### Slope output efficiency of PA and SF lasers

Measured slope efficiencies for 30, 60% $\gamma$ 1O DFB and DBR lasers are given in Table 1.

**Table 1:** Slope output efficiencies for PA, SF 1O DFB and DBR samples for 30, 60% $\gamma$.

Sample	30% $\gamma$ slope output (a.u)	60% $\gamma$ slope output (a.u)
PA 1O DFB	59 ± 12	147 ± 28
PA 1O DBR	272 ± 86	430 ± 139
SF 1O DFB	44 ± 4	33 ± 7
SF 1O DBR	147 ± 47	162 ± 61

In comparing both PA/SF 1O DFB and DBR lasers, significantly higher slope outputs were found in the corresponding DBRs. We attribute this to the fact that the grating is continuous along the length of the DFB cavity and there is decrease in intensity of the resonator mode along the length of the laser due to continuous back-reflection, whereas in DBRs, reflection only occurs at the mirror facets. We observe higher slope outputs in 60% $\gamma$ lasers compared to 30% $\gamma$ lasers for PA samples. The higher output is consistent with a higher overlap of the optical mode with the gratings at higher PDMS fill factors (Confinement 0.064 with 30% $\gamma$ compared to 0.077 with 60% $\gamma$, Table. S1) and reduced grating height of low $\gamma$ PDMS. Additionally, as previously mentioned, the outcoupling from 2nd order Bragg gratings occurs via first order diffraction, we therefore expect the output efficiencies to correlate with first order coupling coefficients, that is, higher outcoupling closer to 50% $\gamma$, which is consistent with the higher slope output with 60% $\gamma$ gratings relative to 30% $\gamma$. For SF lasers, the discrepancy in output was less discernible. In SF DFB lasers, the lower slope output is consistent with lower outcoupling loss, thus lower lasing thresholds, while for DBR lasers the slope output remains comparable within the error margin.

We find substantially higher output efficiency in PA lasers compared to SF for 30 and 60% $\gamma$. A similar increase in efficiency for top-layer gratings was found by Quintana et al*.*^15^ in comparing DCG-air (index 1.55–1) gratings defined above the active layer, and standard SF/DCG-film gratings with a dye-doped polystyrene (index 1.59–1.46 or 1.59–1.55). They found a 3/20-fold increase to the slope efficiency compared to SF/DCG-film lasers, respectively, and have attributed this primarily to increased grating efficiency due to an increased index contrast. However, several other factors ultimately contribute to the magnitude of the radiated power output from the lasers, as demonstrated in analysis of grating-coupled radiation in GaAs waveguides and lasers by Streifer *et al*^28^. They find a complex dependence of radiative output on grating height, duty cycle, index contrast, grating period and the refractive indices of layers adjacent to the grating layer. It is therefore difficult to attribute changes in slope efficiencies, to a single parameter. Numeric calculations may be warranted, to predict the optimal geometries, to obtain the highest outputs.

## Conclusions and perspectives

Lasing was successfully achieved by conformal contact of a composite PDMS stamp patterned with Bragg gratings to an active layer (F8_0.9_BT_0.1_). In this way, the active gain medium is decoupled from the resonator. The stamp could be repeatedly removed from the active-layer surface to recover lasing after degradation, with repeat usage limited predominantly by particle build up on the stamp surface. Although the stamp tends to peel off after initial contact (after several hours/days) we expect applying a small amount of pressure can help sustain contact with the active layer surface.

The emission behaviour of 1st order DFB and DBR lasers with 2nd order out-couplers (1O DFB and DBR), pure 2nd order DFBs (2O) was explored. PDMS-air (PA) grating lasers showed higher thresholds than substrate–film (SF) lasers for a given duty cycle. These higher thresholds are attributed predominantly, to an approximate threefold reduction of confinement in the grating region $.$ Similar thresholds between PA and SF were observed for DBR lasers. This is attributed to low loss and complete reflection in the 1^st^ order mirrors comprising the 1O DBRs. We find slightly lower thresholds in DBRs relative to corresponding DFB lasers in the PA samples but the opposite trend in SF samples. Slope outputs were explored for 30, 60% $\gamma$ 1O DBR and DFB lasers, where higher outputs were found for PA lasers compared to their SF counterparts. Further study is required to determine the origin of this behaviour.

Improvements to the PA structure can be potentially made by adjusting the grating heights as previous reports have shown^13,28–30^. The limit to grating height would, however, be fundamentally limited by the aspect ratio to which the PDMS can be made before pattern collapse. This can be somewhat overcome, by increasing the rigidity of the PDMS, at the cost of increased brittleness. Additionally, as mentioned previously, an increase to laser cavity length for DFB lasers, decreases the threshold at the cost of increased fabrication time.

Overall, we expect the proposed PA system can help accelerate screening of suitable lasing materials without increased patterning/fabrication cost. The system also opens prospects for potential practical application of optically pumped lasers where lasing can be replenished after degradation upon spatial translation of the PDMS across an active film.

## Methods

### Materials

F8_0.9_BT_0.1_ (ADS233YE) was purchased from American Dye Source. Toluene (99.8% anhydrous) was purchased from Sigma Aldrich. (7–8% vinylmethylsiloxane)–(dimethylsiloxane), platinum divinyltetramethyldisiloxane catalyst, 2,4,6,8-tetramethyltetravinylcyclotetrasiloxane and (25–30% methylhydrosiloxane)-dimethylsiloxane copolymer, hydride terminated were purchased from Gelest Corp. Sylgard 184 kit was purchased from Dow chemicals.

### SiO_2_ substrate grating master fabrication

For all DBR and 1O DFB lasers, 40 periods of 2nd order Bragg gratings were used to outcouple light vertically. For 1O DFB lasers, the 2nd order section was placed in the middle of the 1st order gratings as illustrated in Fig. 1e, where the number of 1st order periods were chosen to produce a resonator of roughly the desired length. In 1O DBR lasers, the 2nd order out-coupler is placed at the cavity edge with 500 periods of 1st order gratings comprising the rest of the mirror, while the mirror on the other side of the cavity is only comprised of 500 periods of a 1st order grating (Fig. 1f). The feedback in 2nd order gratings is accomplished via 2nd order diffraction while the light is diffracted out via 1st order diffraction. For 1st order gratings, feedback is accomplished via 1st order diffraction.

Double side-polished fused silica chips (20 × 20 mm^2^) were cleaned in an ultrasonic bath by acetone and IPA. Between acetone/IPA rinses, the chips were physically rubbed by hand via a microfibre cloth and subsequently rinsed with the respective solvents before blown dry by N_2_. The samples were then treated with low RF power O_2_ plasma (RF: 50 W, O_2_: 50 sccm, pressure: 20 mTorr) for 3 min then CHF_3_/O2 plasma for 1.5 min (RF:125 W, CHF_3_: 45 sccm, O_2_: 1.5 sccm, pressure: 20 mTorr) and finally another O_2_ plasma step for 3 min (RF: 50 W, O_2_: 50 sccm, pressure: 20 mTorr). The purpose of these steps were to descum the surface, smoothen the polished surface to improve adhesion of e-beam resist and then a final plasma clean to remove any passivation polymer formed by the CHF_3_ plasma. We find extensive line collapse during the resist development process if the smoothening step is skipped. All etching was performed in a Plasmatherm Vision reactive ion etching system (RIE). The treated Chips were baked/dehydrated at 180 °C for 5 min before 100 nm ZEP520a (1:1 dilution in anisole) was applied via spin-coating. The chips were subsequently baked at 180 °C for 2 min. Elektra92 (conductive polymer) solution was filtered through a 0.22 µm hydrophilic filter and spun atop the baked samples to yield a ~ 40 nm anti-charging layer. A 125 kV electron-beam lithography system (Elionix ELS-F125) was used to pattern the resist with a beam current of 145 pA (dose between 145 and 230 µC/cm^-2^) After exposure, the chips were developed in o-xylene at room temperature for 1 min before quickly blown dry with N_2_. The chips were treated with a post-development bake at 125 °C for 1 min before etched in CHF3/O2 plasma (RF:125 W, CHF3: 45 sccm, O_2_: 1.5 sccm, pressure: 20 mTorr, Plasmatherm Vision) for 4 min 10 s to etch ~ 60 nm SiO_2_, the etch depth differs slightly depending on the feature aspect ratios. The etched chips were then stripped with 10 min O_2_ plasma (RF: 50 W, O_2_: 50 sccm, pressure: 20 mTorr) and rinsed with IPA. All processes up to this point were performed in a cleanroom.

### Substrate–film sample preparation

For the Substrate–film sample, the chips were baked at 180 °C for 5 min before F8_0.9_BT_0.1_ in toluene (25 mg/mL) was spun-coat as is at 2000 rpm to yield a film of ~ 180 nm (without gratings) and was used as is, without annealing (annealing above the glass transition temperature drastically raises the lasing threshold). The F8_0.9_BT_0.1_ solution was prepared in a N_2_ filled glovebox but spun in ambient conditions.

### PDMS composite stamp fabrication^31^

The SiO_2_ etched samples were used as a master for PDMS replication. The chips were baked at 180 °C for 10 min before being placed in an evacuated desiccator with 7 µL TCOFS on a separate holder for 1 h. A droplet of deionized water was used to test hydrophobicity and the sample was rinsed with IPA afterwards to clean the surface as a milky film tends to deposit on the chips during the TCOFS coating process.

To prepare h-PDMS, a mixture of 0.791 g (7–8% vinylmethylsiloxane)–(dimethylsiloxane) with 7 µL platinum divinyltetramethyldisiloxane catalyst and 24 µL 2,4,6,8-tetramethyltetravinylcyclotetrasiloxane modulator was made. To this, 230 µL (25–30% methylhydrosiloxane)-dimethylsiloxane copolymer, hydride terminated was added along with 540 µL toluene. Toluene is used to provide better moulding of the mixture into the trenches of the patterned nanostructures^32^. The mixture was then quickly degassed with a vacuum desiccator and poured over the master chip and spun at 1000 rpm for 60 s. The sample was left to rest for 1 h in ambient conditions before baked in an oven at 60 °C for 10 min.

To prepare the soft PDMS, Sylgard 184 base was mixed with its curing agent in a 9:1 weight ratio, stirred thoroughly and degassed in vacuum, the mixture was poured over the h-PDMS covered chips in a petri dish and subsequently degassed in vacuum again. The resulting samples were then cured in an oven at 70 °C for 5 h, cooled, and left resting in ambient conditions for more than 12 h before the sample was removed from the petri dish and the master chip removed with a scalpel and then peeled off. The resultant stamp is cut at the edges to remove any large protrusions that may prevent conformal contact with lasing active film.

### PDMS-air laser preparation

A 30 × 30 mm^2^ fused silica chip was cleaned following the steps in the SF/SiO_2_ master fabrication including the plasma cleaning steps. The chip was baked at 180 °C for 5 min before F8_0.9_BT_0.1_ in toluene (25 mg/mL) was spun coat at 2000 rpm to yield a film of ~ 180 nm and was used as is, without annealing. The F8_0.9_BT_0.1_ solution was prepared in a N_2_ filled glovebox but spun in ambient conditions. The PDMS stamp was placed atop the film and gently pressed until conformal contact was made.

### Optical characterization

Lasing measurements were carried out using the output from a diode-pumped, active Q-switched frequency tripled Nd: YVO_4_ (1.1 ns) laser (Picolo MOPA, Innolas) at 355 nm. The repetition rate was changed between different samples depending on the signal obtained, for higher output signals, the repetition rate was decreased to prevent saturation of the camera while running in continuous acquisition mode. However, signals are all scaled to a 10-pulse signal for slope output efficiency measurements. The fabricated samples were mounted on an xyz stage and were excited at normal incidence with an ~ 200 µm × 50 µm stripe formed by a set of optics (Fig. S7). The pump light was filtered out via a long-pass filter with the output emission is collected at normal incidence, directed with a set of mirrors, and focused onto the entrance slit of a spectrograph comprised of an Acton 2150i spectrometer (15 mm focal length) and an sCMOS camera (PCO edge 3.1). For zero order diffraction measurements, the entrance slit was fully opened, while for lasing and spectral characterization, the slit is set to 50 µm, resulting in a spectral resolution of ~ 0.7 nm.

### Numeric mode-solver calculations

Mode calculations were performed in Mode solutions (Lumerical) based on a finite-difference eigen solver (FDE) method at 565 nm for the fundamental TE_0_ mode. Parameters used are SiO_2_ = 1.46, F8_0.9_BT_0.1_ = 1.7, Air = 1, PDMS = 1.43.

## Supplementary Information

Supplementary Information.

## Data Availability

The datasets generated during and/or analysed during the current study are available from the corresponding author on reasonable request.
