# Supplementary material for: Active waveguide Bragg lasers via conformal contact PDMS stamps
Source: Sci Rep. 2022 Dec 23;12:22189. doi: 10.1038/s41598-022-26218-7 (PMC9789066; doi:10.1038/s41598-022-26218-7)
Supplement: Supplementary file 1 — Supplementary Information. [file 41598_2022_26218_MOESM1_ESM.pdf]

Supporting Information

Active Waveguide Bragg Lasers via Conformal Contact PDMS Stamps

Yun Li,[1] Girish Lakhwani[1-3] *

[1] ARC Centre of Excellence in Exciton Science, School of Chemistry, University of Sydney. NSW 2006, Australia

[2] The University of Sydney Nano Institute, Sydney, NSW 2006, Australia

[3] Institute of Photonics and Optical Science, The University of Sydney. NSW 2006, Australia

*Author to whom correspondence should be addressed: girish.lakhwani@sydney.edu.au

SEM and AFM images of SiO_2 and PDMS Gratings of Varying Duty Cycle

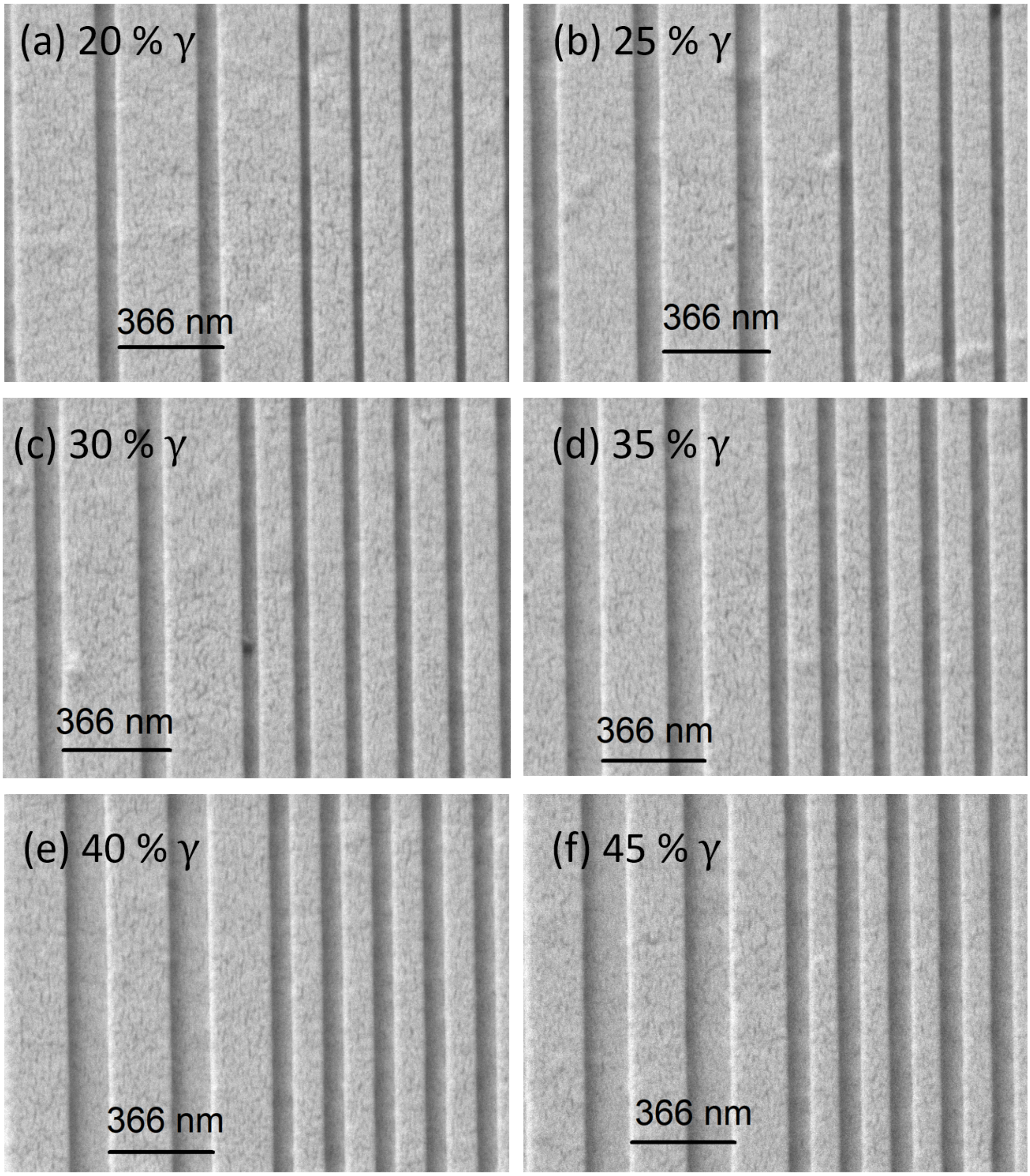

Figure S1. SEM of SiO_2 gratings (a-f) for varying pattern duty cycles (γ) between 20-45% with gold anti-charging layer for imaging.

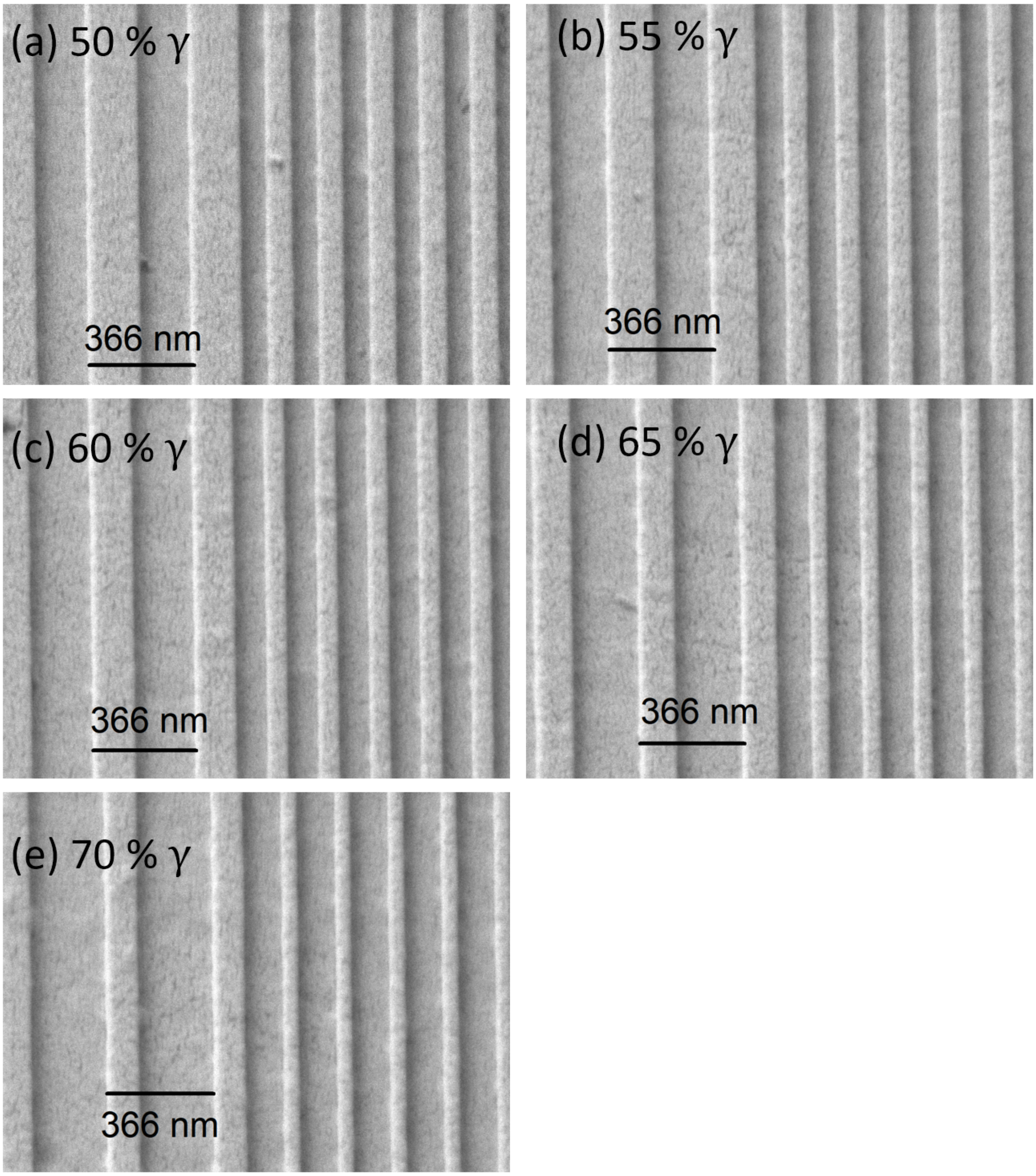

Figure S2. SEM of SiO_2 gratings (a-e) for varying pattern duty cycles (γ) between 50-70 % with gold anti-charging layer for imaging.

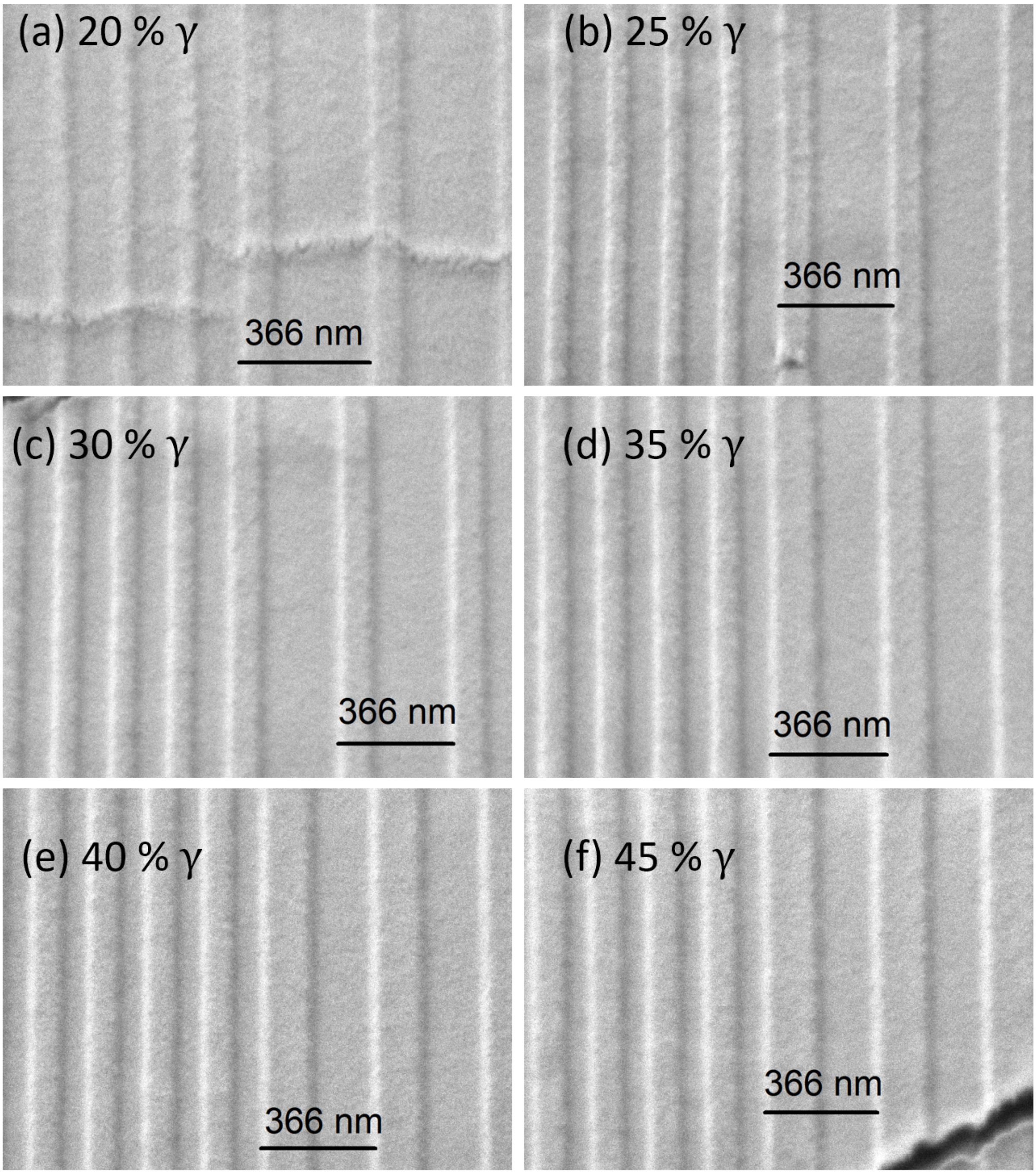

Figure S3. SEM of PDMS gratings (a-f) for varying pattern duty cycles (γ) between 20-45 % with gold anti-charging layer for imaging.

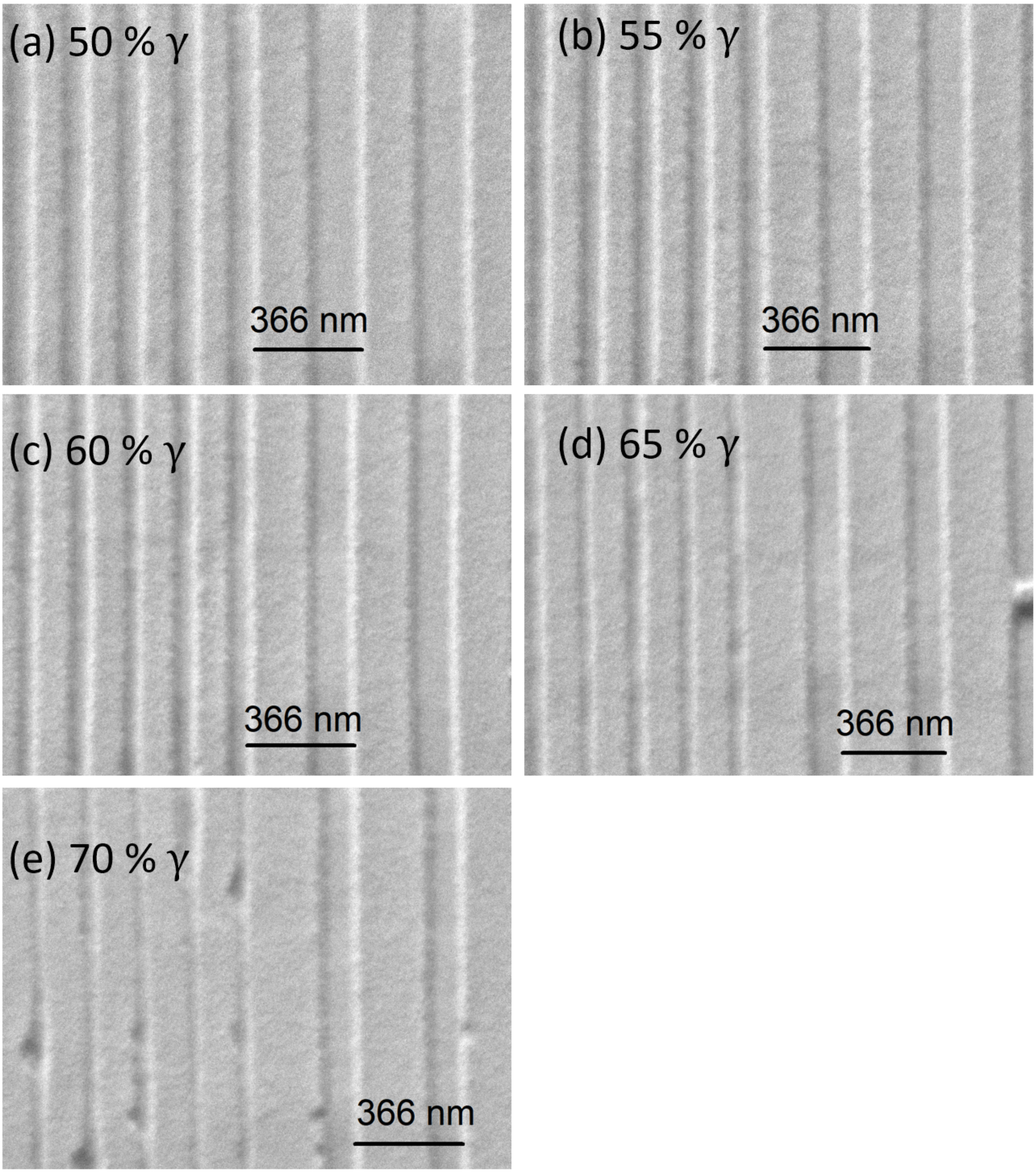

Figure S4. SEM of PDMS gratings (a-e) for varying pattern duty cycles (γ) between 50-70 % with gold anti-charging layer for imaging.

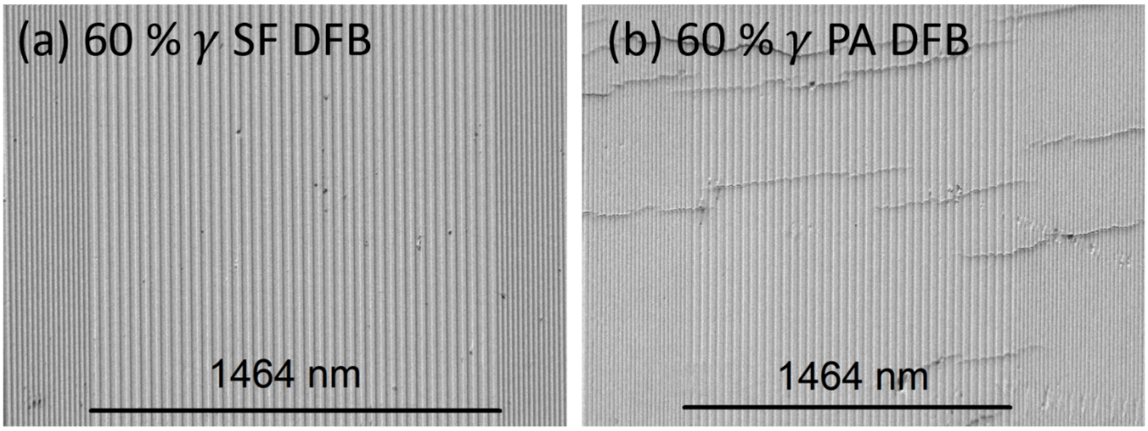

Figure S5. 60 % γ 1O DFB patterns showing 40 2nd order periods sandwiched between 1O gratings for a) SF, and b) PA samples.

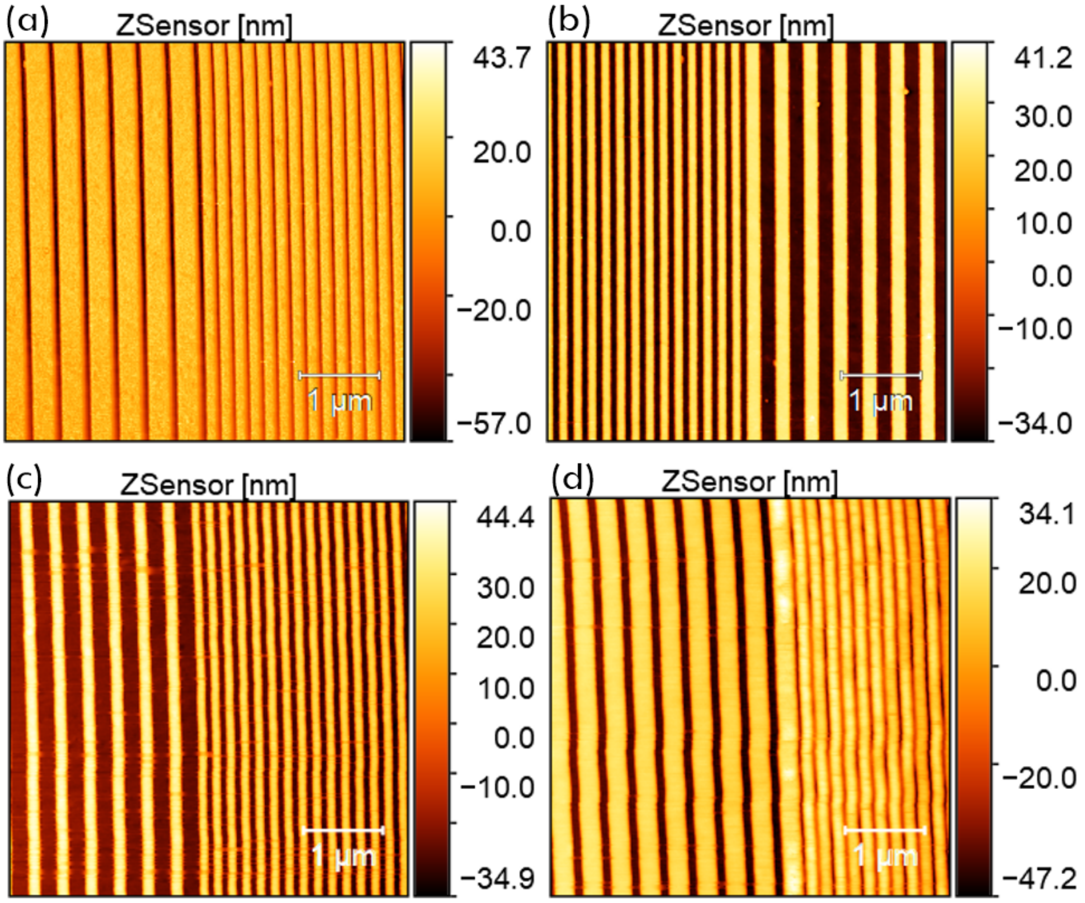

Figure S6. AFM for a) SF 30, b) SF 60 c) PA 30, d) PA 60 % γ gratings.

Note in Fig. S6 for high aspect ratio gratings, the AFM tip is unable to acquire accurate depth profiles due the relatively large tip size.

Optical Characterization

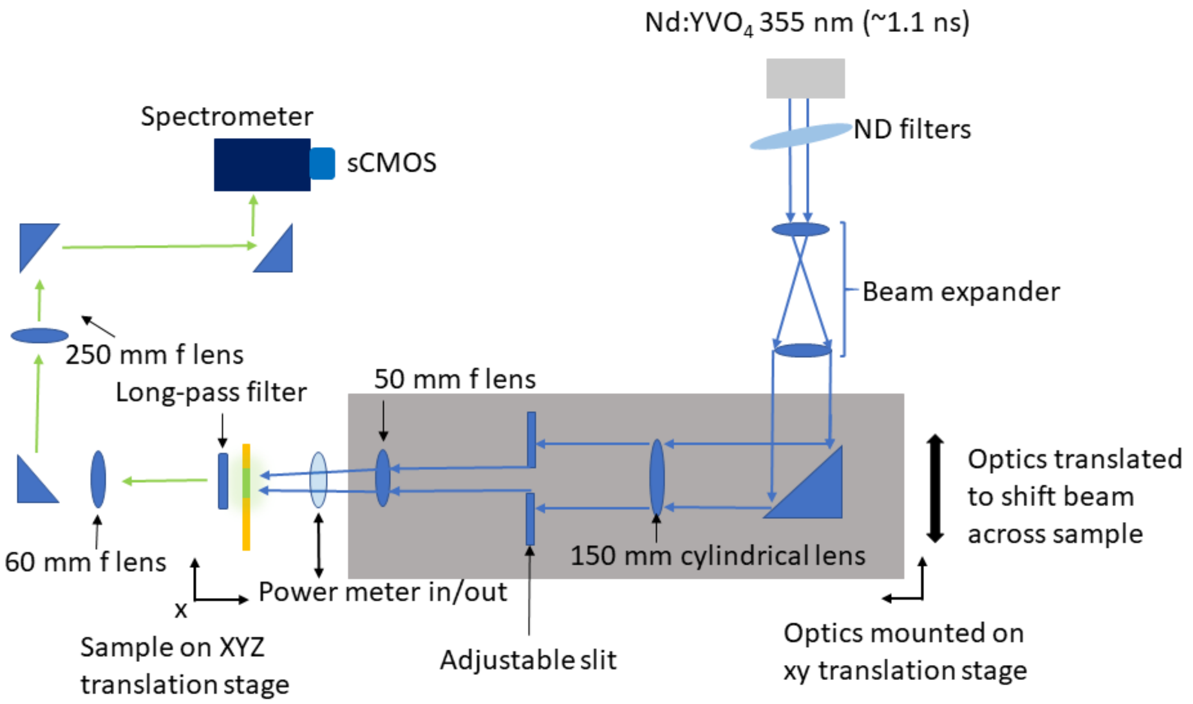

Figure S7. Experimental setup for lasing measurements.

The experimental setup is illustrated in Fig. S7. A set of ND filters was used to adjust the incident power density upon the sample, while a set of optics was used to shape a 200 x 50 μm^2 beam. The incident average power was measured by a S120VC (Thorlabs) power meter inserted directly in the path of the incident beam just before the pump beam is incident on the sample and taken out when a measurement was taken. The pump beam was filtered out by a long pass filter (420 nm), and the lasing output imaged/focused onto the entrance slit of a spectrograph by 60 mm/250 mm lenses. The spectrograph is comprised of an sCMOS camera (PCO edge 3.1) run in global shutter; continuous-acquisition mode attached to an Acton 2150i spectrometer with 15 mm focal length. In this configuration, the image of the sample plane was roughly in focus and magnified by ~3.8x at the spectrograph entrance slit/back focal plane (assuming 1:1 imaging from the entrance slit). This magnification factor is calculated by comparing the beam length (200 μm) as shown in Fig. S8(a-c) and the 0th

order diffraction images on the spectrograph in Fig. S9(a, c, e). Each physical pixel in the camera is 6.5 µm and is binned in the x-dimension by 2x, so the effective pixel is 13 µm, the image spans ~ 57 pixels, so the image magnification factor is 57x13 µm /200 µm = 3.8.

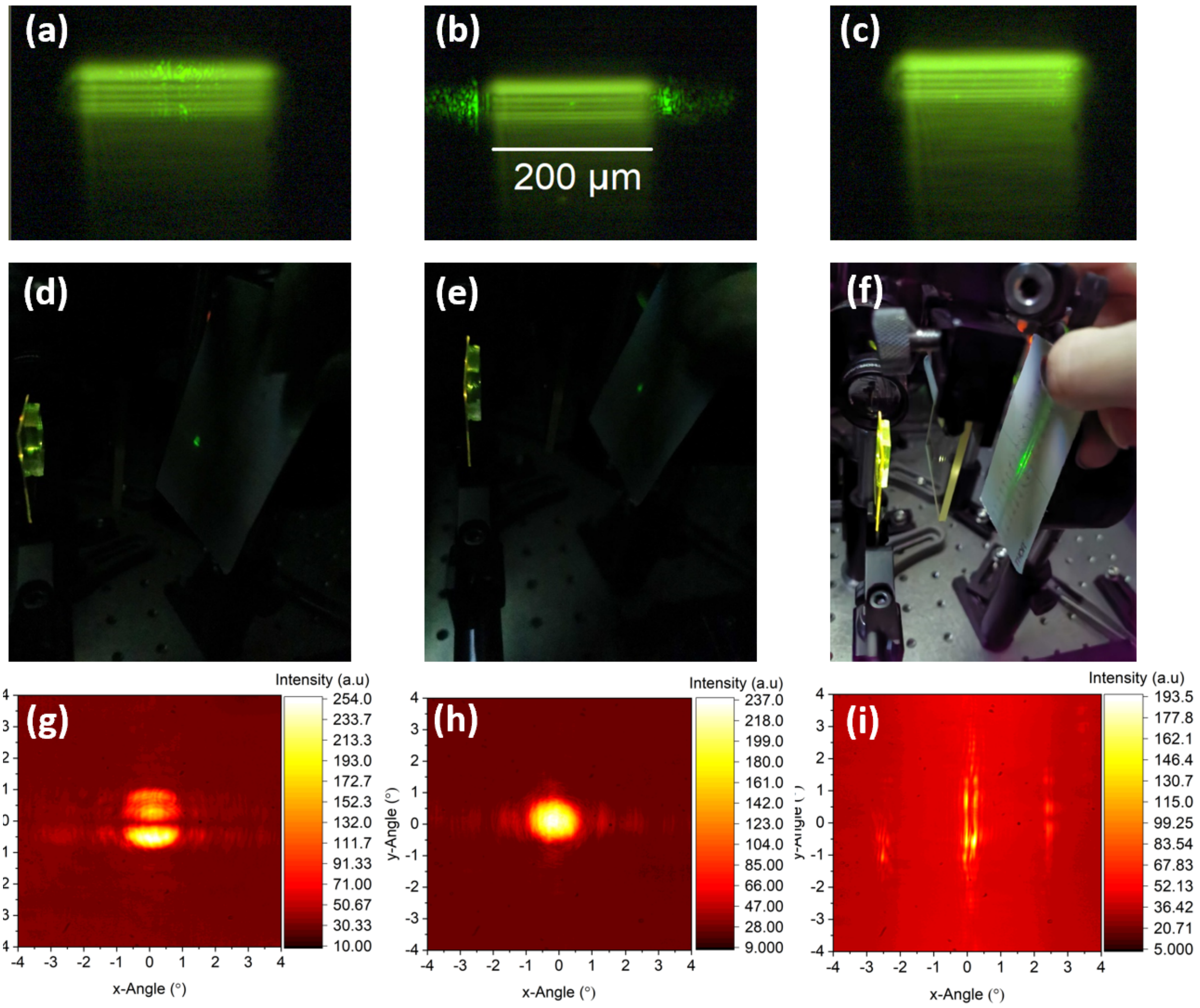

Figure S8. Zoom lens images of 60 % γ PA a) 1O DFB, b) 1O DBR, c) 2O DFB lasers, and their respective photo-camera images d), e), f) of the far-field emission and g), h), i) camera images of far-field taken at ~12 mm from sample with approximate angles.

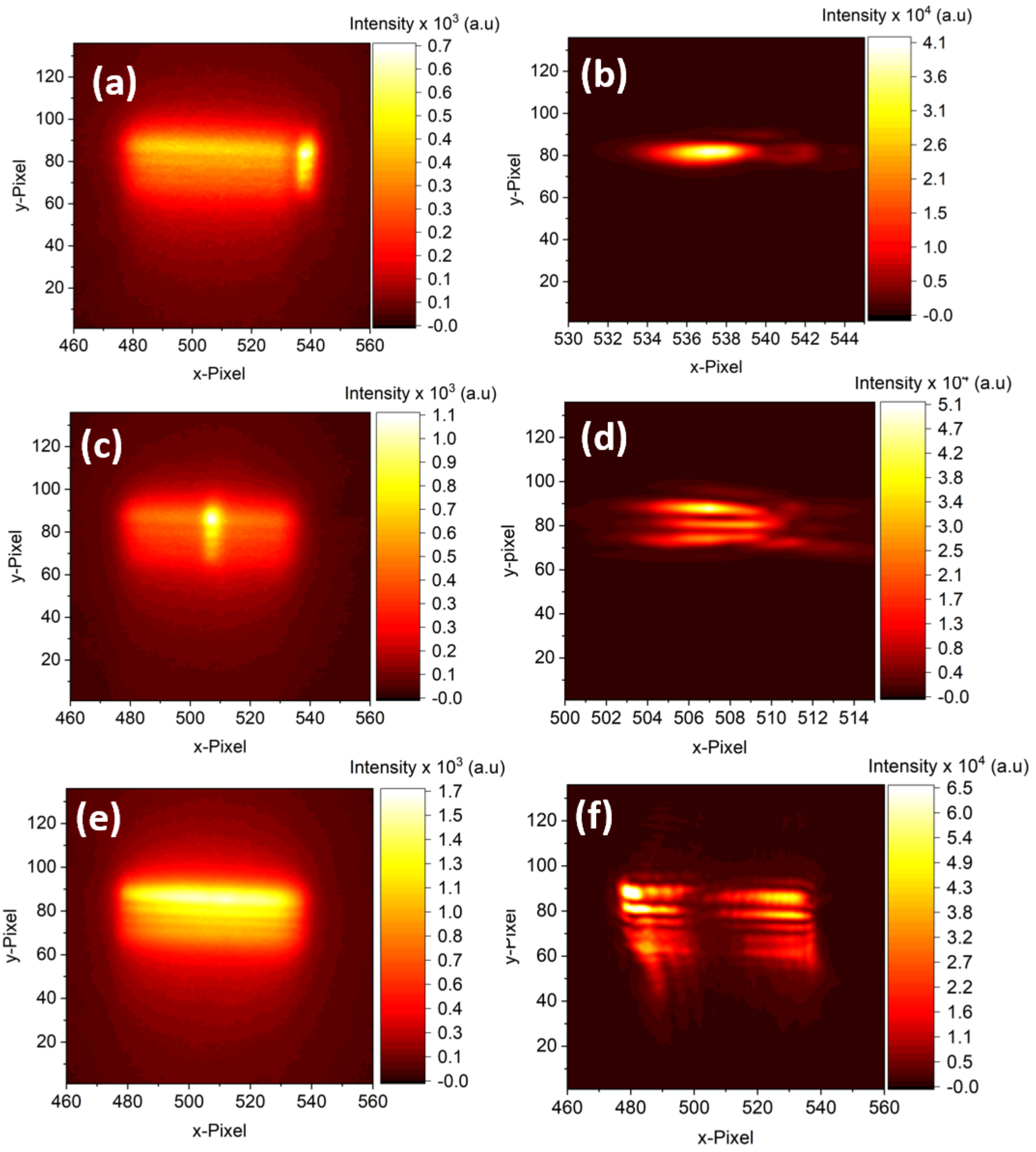

Figure S9. Magnified (~4x) "Near-field" images captured with fully open slit spectrograph in 0th order diffraction (reflection mode) where a), c), e) and b), d), f) are pre and post lasing threshold respectively. Samples are a), b) 1O DBR, c), d) 1O DFB and e), f) 2O lasers.

Scattering from mirror facet edges was observed in 1O DBR samples (Fig. S8(b)). Far-field (FF) images were taken with a Sony IMX317 CMOS USB camera (pixel size 1.6 μm x 1.6 μm), ~12 mm from the laser source. Angles given in Fig. S8(g-i) are determined with simple trigonometry and only apply to the central axis cross-sections. Divergence angles of <1° was found for all samples. We observe the characteristic null zone in intensity for the

2O DFB[1] in Fig. S8(f, i) and Fig. S9(f) for FF and nearfield profiles respectively. The null arises due to destructive interference of radiation against counter-propagating coupled waves in the central region resulting in the null in scattered radiation.

Mode-Solver calculations

Mode calculations were performed in Mode solutions (Lumerical) based on a finite-difference eigen solver (FDE) method at 565 nm for the fundamental TE_0 mode. For SF samples, semiconductor thickness was swept as shown in Fig. S10(a), to observe the variation in confinement 60 nm into the active film and 60 nm into the SiO_2 substrate, and confinement in the active semiconductor layer. Also plotted is the effective refractive index. The semiconductor thickness was swept to observe changes in confinement as spin-coating smoothens out the active film and the regions with SiO_2 corrugations will have corresponding lower confinements. For PA gratings, the air thickness is swept as shown in Fig. S10(b).

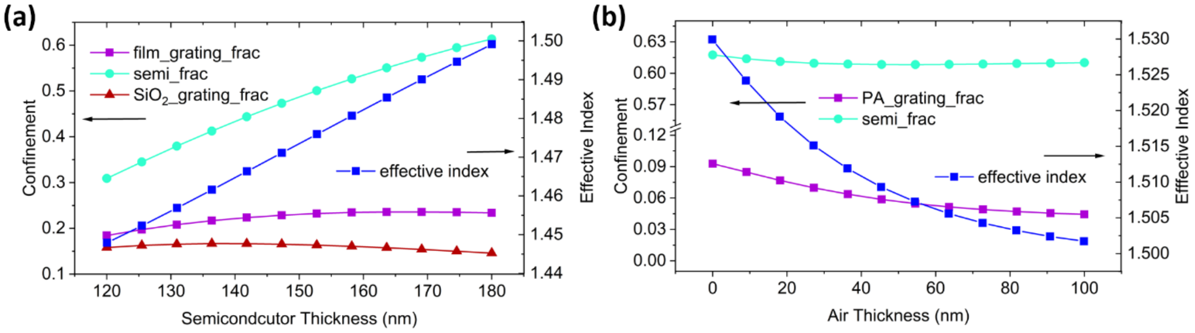

Figure S10. Mode solver calculations of planar waveguide stack (SiO_2 = 1.46, $F8_{0.9}BT_{0.1}$ = 1.7, Air = 1 , PDMS = 1.43 @ 565 nm) for TE_0 mode of a) SF sample with semiconductor layer (active layer) thickness swept and showing effective refractive index, confinement in the active film (semi_frac), confinement 60 nm above the substrate (or 60 nm into the active layer) (film_grating_frac) representing the mode overlap with the film portion of the grating, and confinement 60 nm below the substrate (SiO_2_grating_frac) representing mode overlap with the SiO_2 portion of the grating. (b) PA sample with air thickness (between

active layer and PDMS) swept and showing effective refractive index, confinement in the active layer and confinement within the swept air layer.

Multi-mode Lasing in DBR and DFB Lasers

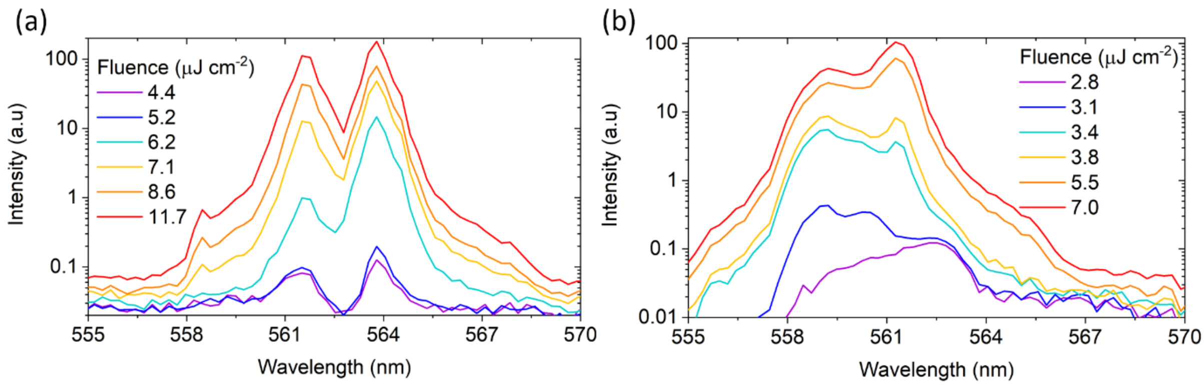

Figure S11. (a) 60 % γ PA 1O DFB with growth of two lasing peaks at each side of the photonic stopband, and (b) with mode hopping and spectral instability within the reflection band of a 30 % γ SF 1O DBR laser.

Lasing from both sides of the photonic stopband was observed in Fig. S11(a) due to a lack of mode selectivity mechanism. DBR mode hopping due to potential multiple resonances lying within the reflection band shown in Fig. S11(b). The individual modes are smeared out as the spectra was averaged; however, clear mode-hopping is observed if taken frame by frame.

Fluence-dependent spectra for PA 60 % γ 2O and 1O DBR lasers

Fluence-dependent spectra for PA 60 % γ 1O DBR and 2O lasers are illustrated in Fig. S12(a) and (b) respectively.

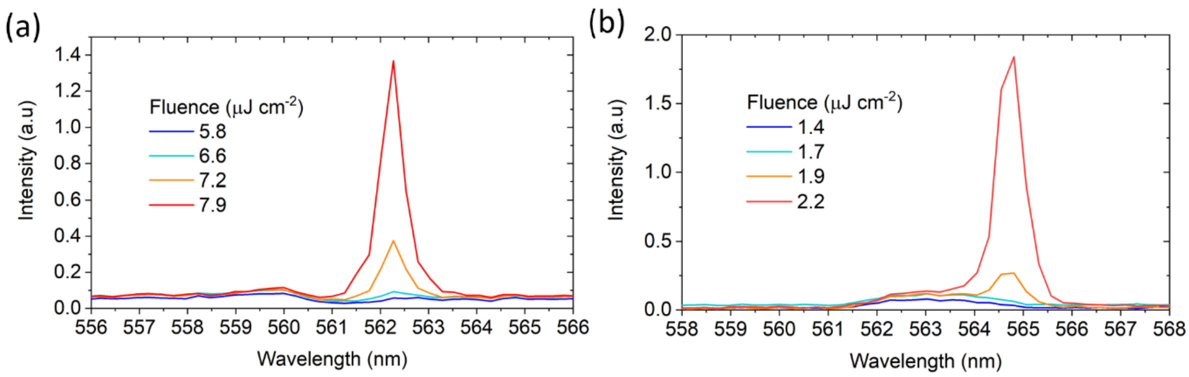

Figure S12. Fluence-dependent spectra for 60 % γ PA a) 2O DFB and (b) 1O DBR.

Overcoating of Active Film Over Substrate-film Gratings

AFM of the film overcoating on SiO_2 gratings (SF sample) 60 % γ is shown in Fig. S13. The corrugations were dramatically smoothened and a height modulation of ~ 8 nm and ~2 nm is found for 2nd and 1st order gratings respectively. Cross sectional images of the overcoated film is depicted in Fig. S14. Observed is a relatively flat interface with the gold anti-charging layer, corroborating the corrugation smoothening observed in Fig. S13.

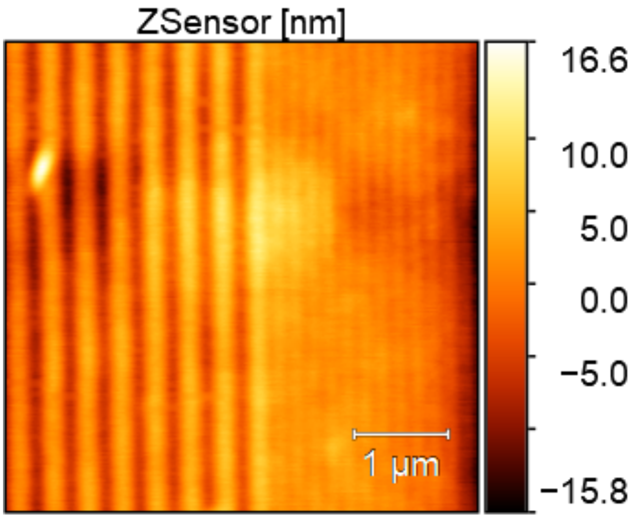

Figure S13. Overcoating of active film over SiO_2 gratings for 60 % γ samples. Roughly 8 nm corrugation in the 2nd order section and <2 nm in the 1st order section. Accuracy is subject to tip bluntness and feature aspect ratio.

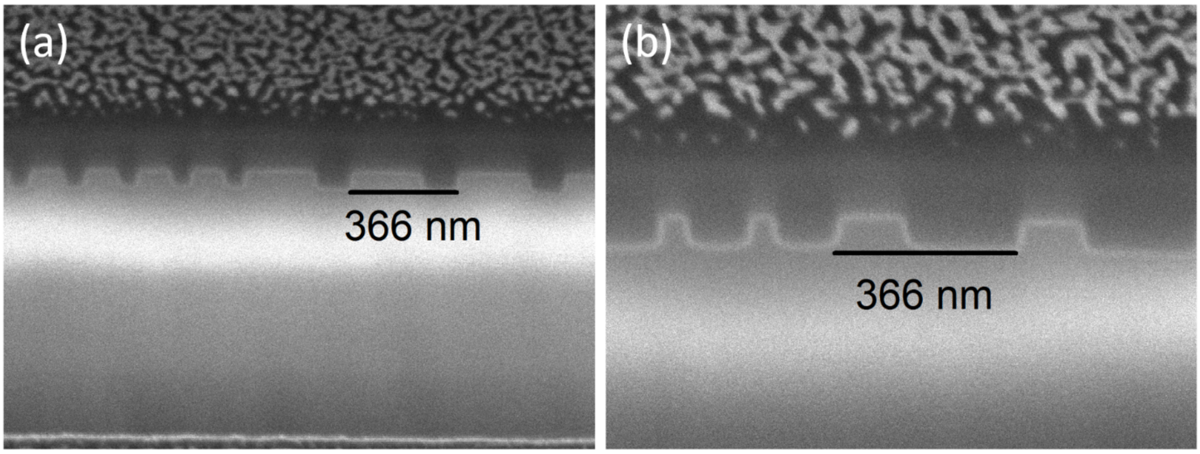

Figure S14. SEM cross section of (a) 30, (b) 60 % γ SF with a sputtered gold conductive layer overcoating to reduce charging. Image distortion presented as a "shadow" due to charging, otherwise the films are seen to be relatively flat. Note that this is not the sample used in the main text, but a separate sample for illustrative purposes; due to electron-beam lithography over-exposure, the trenches are wider than expected and of that used in the main text.

TM$_0$ Mode Observation

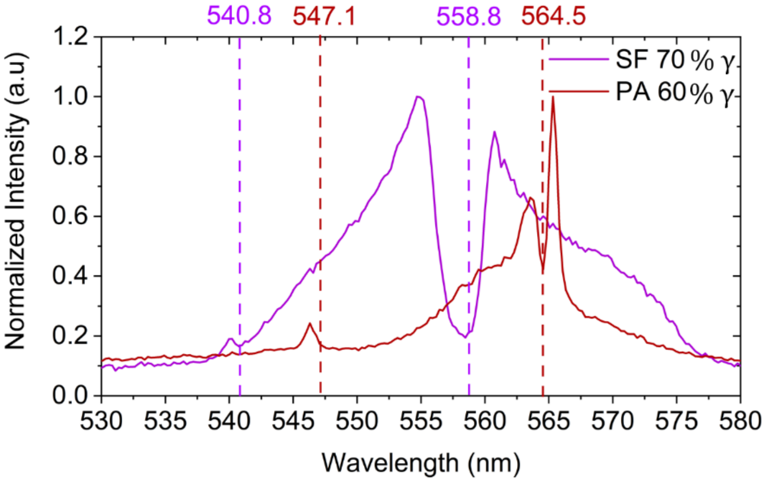

Figure S15. Observation of Bragg dip from TM$_0$ mode.

A small dip is observed approximately 18 nm from the TE$_0$ stopband as shown in Fig. S15. This is assigned to the TM$_0$ mode based on comparison of the numerically calculated

TM$_0$ and TE$_0$ n$_{eff}$ and their predicted relative Bragg wavelengths in Lumerical's mode-solver. The exact difference in n$_{eff}$ depends on the active layer thickness and air thicknesses as illustrated previously in Fig. S10, however, the resultant difference in spectral position between TM$_0$ and TE$_0$, is estimated to be at-least 10 nm in most cases.

Spectra for varying duty cycle SF and PA 1O lasers

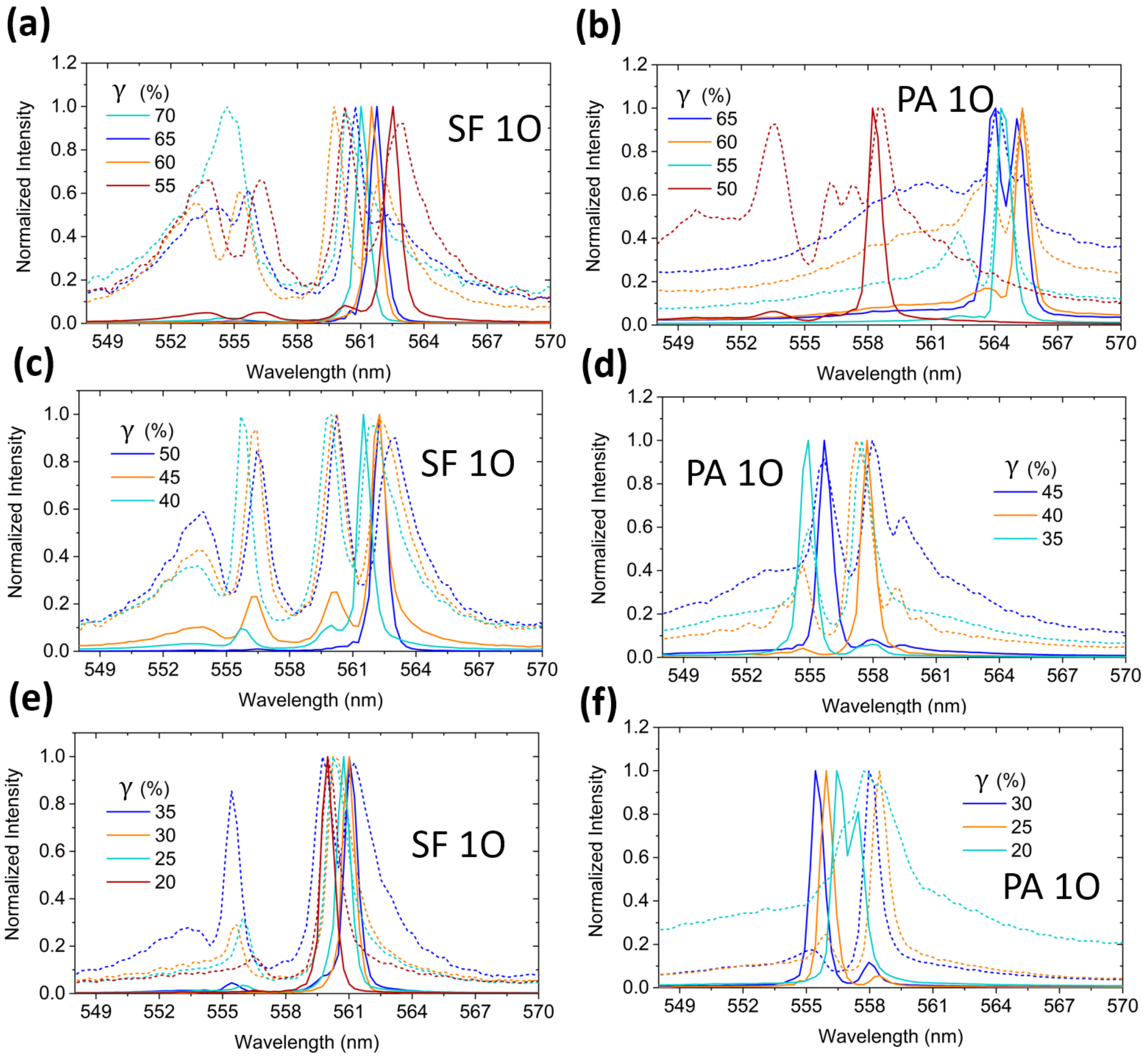

Figure S16. Emission spectra below (dashed lines) and above lasing (solid lines) threshold for PA and SF 1O DFB samples of different duty cycles with (a) SF 70-55 % γ, (b) PA 65-50 % γ, (c) SF 50-40 % γ, (d) PA 45-35 % γ, (e) SF 35-20 % γ, (f) PA 35-20 % γ.

Spectra for 1O DFB lasers varying γ are given in Fig. S16. In Fig. S16(a), the photonic stopband is clearly identified for 70 % γ SF laser. Towards 55 % γ, the band-edge peak appears to form a dip in the spectra. In Fig. S16(e), a high intensity band-edge peak can be identified on the long wavelength edge of the stopband for γ below 35 %. At 35 % γ, a dip emerges at both band-edges. The emergence of the dips is accompanied by a reduction of the high-wavelength band-edge peak intensity relative to the low wavelength edge as evident in the normalized spectra. In all cases where these dips form, lasing appears on the high-wavelength side of the dip.

Grating Period-dependent Spectra

Clear spectral shifts in lasing wavelength were observed with adjusted grating periods as shown in Fig. S17(a), consistent with the Bragg equation. Preferential lasing on the low energy edge of the photonic stopband was observed for all periods except for 185 nm 1st order periodicity, where lasing occurs on both longitudinal modes. Note that the laser thresholds in Fig. S17(b) for 1O samples were slightly lower than reported in Fig. 4(f) due to a mixed γ configuration (50% γ 1st order grating and a 75% γ 2nd order grating), thus minimizing the lasing threshold by maximizing the feedback/coupling coefficient.

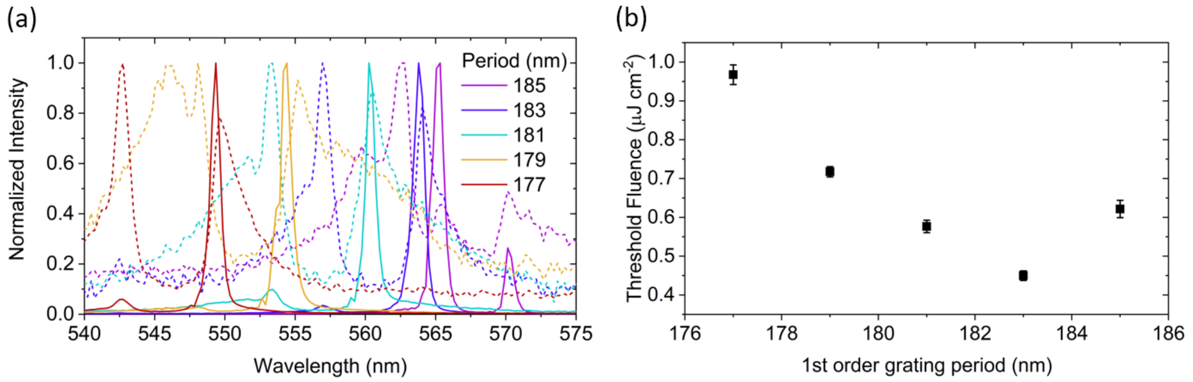

Figure S17. (a) Normalized Spectra for SF 1O DFB emission with a 75 % γ 2nd order grating and 50 % γ 1st order grating with dotted/closed lines corresponding to below/above threshold

for different 1st order grating periodicities (2nd order out-coupler is double the grating period of 1st order) (b) threshold fluence as a function of 1st order grating periodicity.

Cavity length dependent threshold

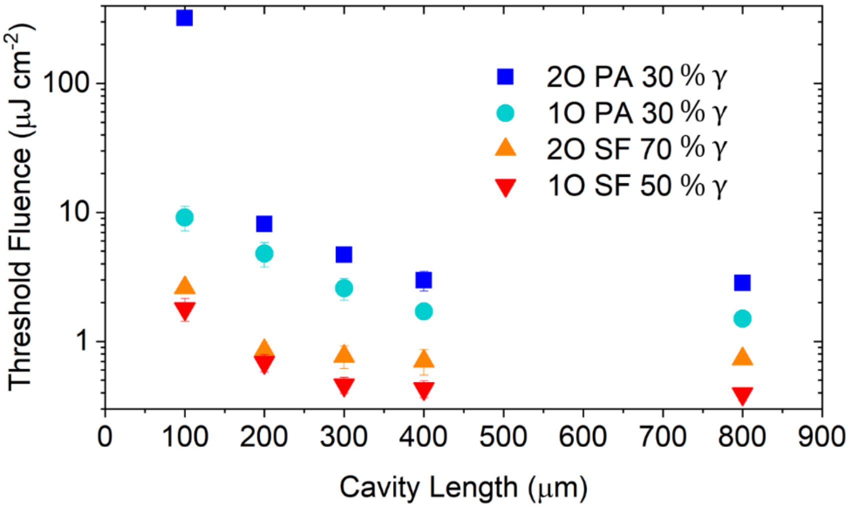

Figure S18. Cavity-dependence of lasing threshold fluence for PA 30 % γ 2O/1O and SF 50 % γ 1O and SF 70 % γ 2O DFB lasers.

A significant decrease in threshold fluence was observed for longer cavity lengths due to higher total feedback as shown in Fig. S18. However, the relative reduction in threshold tapers off towards larger cavity lengths. Saturation in threshold occurs at longer cavity lengths in PA lasers compared to SF lasers as a result of lower κ, as predicted by coupled-wave theory of DFB lasers[2]. Overall, the thresholds in PA lasers were consistently higher than that of SF lasers, where γ was chosen close to the lowest thresholds obtained for each respective type of laser from Fig. 4(f) at 200 µm cavity lengths.

Calculation of Confinement in Grating Region

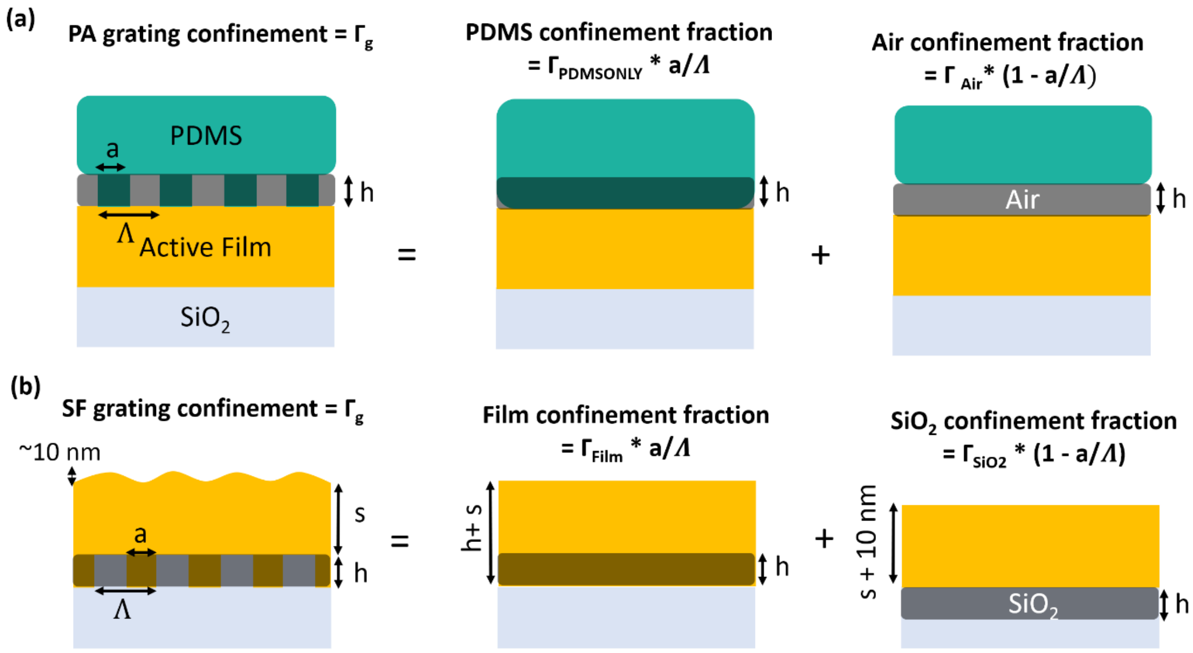

Figure S19. Schematic for grating confinement calculations of (a) PA, (b) SF samples. Shaded region is the area taken for integrated optical power (optical confinement), h = 60 nm.

Calculations for the grating confinement is illustrated in Fig. S19. For PA gratings, the grating confinement was calculated by calculating the confinement in the PDMS section of the grating and then the air section separately as planar waveguides. We then take the sum of the two modulated by the fill factor or duty cycle. For SF gratings, we assume the film section will be modulated by 10 nm due to a h = 60 nm grating. For a 180 nm film, we assume s = 180 - h = 120 and the film will be 120 + 10 = 130 nm, and the confinement was calculated 60 nm into the SiO_2 substrate. For the film section, we assume no modulation from the grating and calculate the confinement 60 nm into the active film from the SiO_2-film interface.

Confinement is defined as expressed in Eqn. S1,

$$\Gamma = \frac{\iint_{grating\ area} |U|\ dA}{\iint_{\infty}^{\infty} |U|\ dA}, \qquad (S1)$$

Where U is the modal field distribution and A is the grating area, enclosed by grating height h. We calculate average effective indices in a similar way to the confinement here.

Table S1. Parameters defined in main text for 30, 60 % γ PA and SF samples.

Sample	30 % γ PA	60 % γ PA	30% γ SF	60% γ SF
$\frac{a}{\Lambda}$	0.3	0.6	0.3	0.6
n_{eff}	1.523	1.516	1.485	1.471
Γ_g	0.064	0.077	0.184	0.206
n_2	1.43	1.43	1.7	1.7
n_1	1	1	1.46	1.46
$\sin\left(\frac{\pi a}{\Lambda}\right)$	0.81	0.95	0.81	0.95
$\sin\left(\frac{2\pi a}{\Lambda}\right)$	0.95	0.59	0.95	0.59

Loss Measurements

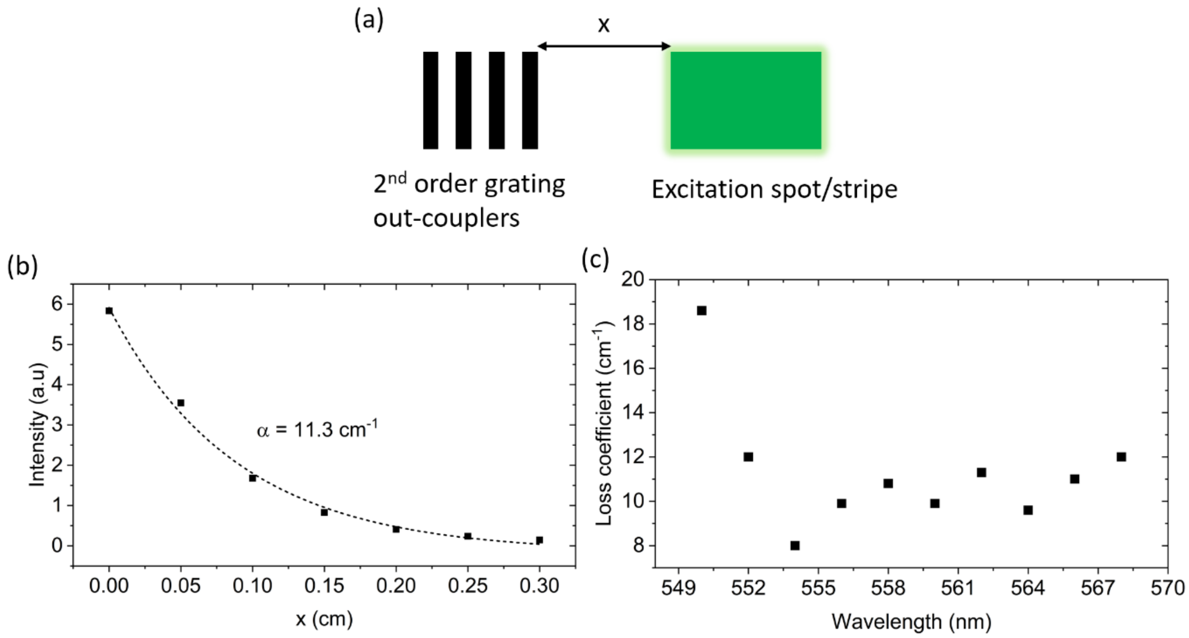

Figure S20. (a) Schematic of measurement used for measuring loss using a 2nd order grating out-coupler on SF sample with, (b) standard exponential fit to loss @ 562 nm, c) spectral dependent loss.

The schematic for measuring waveguide loss is shown in Fig. S20(a). Here, a 2nd order grating was used to outcouple waveguided light from a 200x50 µm excitation stripe. The out-coupler section is imaged onto the entrance slit of the spectrograph with the excitation stripe blocked out by the slit. The excitation stripe is translated away from the grating and attenuated with distance x. The loss was fit to a simple exponential function $y = e^{-\alpha x}$ as shown in Fig. S20(b) with wavelength dependence loss given in Fig. S20(c). Similar losses are found using both PA and SF gratings.

References

[1] Henry, C., R. Kazarinov, R. Logan, and R. Yen, *Observation of destructive interference in the radiation loss of second-order distributed feedback lasers.* IEEE Journal of Quantum Electronics, 1985. **21**(2): p. 151-154.

[2] Kogelnik, H. and C. Shank, *Coupled-wave theory of distributed feedback lasers.* Journal of applied physics, 1972. **43**(5): p. 2327-2335.
